# Media consumption and self-perceived multilingual identity in short-video vlog-assisted informal digital language learning

**DOI:** 10.3389/fpsyg.2026.1915495

**Published:** 2026-07-15

**Authors:** Yuhang Ren, Xin Wang

**Affiliations:** 1 INTI International University, Nilai, Malaysia; 2 School of Journalism and Communication, Chongqing University, Chongqing, China

**Keywords:** digital literacy, informal digital language learning, learning engagement, media consumption (communication), multilingual identity, short video vlogs, social presence

## Abstract

**Background:**

Short video vlogs have become a prominent site of informal digital language learning, yet most research has emphasized skills, motivation, and willingness to communicate, with comparatively little attention to social affective and identity related self-perceptions. This study examined how short video vlog assisted informal digital language learning is associated with self-perceived multilingual identity, whether social presence and learning engagement are modeled as serial intermediate variables, and whether digital literacy conditions these associations.

**Methods:**

A cross sectional questionnaire was completed by Chinese university language learners (*N* = 648). A serial indirect-association model and a moderated conditional-process model were estimated with 5,000 bootstrap resamples, together with robustness and sensitivity analyses.

**Results:**

Vlog assisted informal learning was positively associated with self-perceived multilingual identity both directly and indirectly through social presence and learning engagement, and the serial indirect association running from social presence to learning engagement was significant. Digital literacy moderated all three associations originating in vlog learning, each being stronger at higher levels of digital literacy, and every index of moderated mediation was significant.

**Conclusion:**

Everyday vlog based informal practice is associated with self-perceived multilingual identity to the extent that the environment is experienced as socially present and engaging, and to the extent that learners possess the digital literacy to act on the affordances such environments make available. The findings extend social presence, engagement, and self-perceived multilingual identity scholarship into informal short video settings and underscore the value of careful data screening in survey research on digital learning.

## 1 Introduction

For a growing number of young people, the most frequent encounters with other languages now happen on commercial short video platforms, in the unstructured, interest driven feeds where they watch, scroll, comment, and post (Sauro and Zourou, 2019). Language learning has, in effect, moved onto algorithmically curated entertainment services that were never designed for instruction, where a single vlog fuses personal narration, everyday speech, visual storytelling, platform interaction, and cultural self presentation, and circulates through feeds, comments, danmu, and follower networks that make using a language a social act (Li and Ding, 2026; Ou et al., 2024; Sun et al., 2026). This migration poses a pressing question for educators and platform designers alike: when everyday exposure to multilingual content takes place through short video feeds, is such exposure associated with young people’s secure, integrated sense of themselves as multilingual speakers, and is that association evident across learners, or only those equipped to make use of what these platforms afford (Palacios-Hidalgo and Huertas-Abril, 2025; Shi and Xu, 2025)? The question carries an equity stake: if the identity related relevance of informal digital exposure depends on capabilities that are unevenly distributed, the same feeds may leave some learners further behind (Guamos and Estremera, 2025). Understanding what learners take from these environments, and under what conditions, is therefore central to contemporary research on language, media, and the self (Arndt, 2023; Shi and Xu, 2025).

Research on digitally mediated language learning has moved through a recognizable trajectory. An initial wave treated social media as a language learning environment and catalogued the affordances, including interactivity, collaboration, authenticity, multimodality, persistence, and networkedness, that distinguish networked platforms from the classroom (Barrot, 2022; Guamos and Estremera, 2025). A second wave turned from platforms to practices, reframing out of class digital activity primarily as informal digital learning of English (IDLE), and documenting its links to vocabulary, speaking, motivation, enjoyment, and self efficacy (Lee, 2019; Lee and Dressman, 2018). A third, more recent wave has become tool specific, isolating particular genres of informal practice: vlog assisted informal language learning has been conceptualized and measured along three dimensions, content consumption, interactive participation, and language application, that move beyond the broad receptive and productive dichotomy of earlier IDLE work (Sun et al., 2026; Zou et al., 2025). In parallel, studies of learner generated vlogs have shown that vlogging is also a site of self presentation, social visibility, and imagined future selves, beyond serving as a source of input (Li and Ding, 2026). Across these developments, the field has shifted its attention from what platforms offer toward what learners actually do with what is offered.

That shift exposes the theoretical problem at the heart of this study. From an ecological perspective, a digital environment is best understood as a field of affordances, representing action potentials and opportunities for educational activities that emerge from the dynamic interaction between learners and their environment, which can be facilitative or inhibitory depending on what the learner perceives and acts upon (Jiang and Zhang, 2019; Xue and Churchill, 2019). Affordances are not themselves identity-relevant experiences: they become salient only as learners exercise agency to perceive, interpret, and actualize them (Jiang and Zhang, 2019; Li and Ding, 2026). Learner generated vlogging, for example, is organized around linguistic, digital, social, and emotional affordances that learners take up unevenly (Li and Ding, 2026). Exposure to multilingual vlog content cannot, on its own, be assumed to correspond to the focal identity related construct at stake here, multilingual identity, understood as a relational, socially positioned, and continually negotiated sense of self (Bucholtz and Hall, 2005; Darvin and Norton, 2015; Fisher et al., 2018). The pertinent questions are accordingly associational and conditional: through what experiences is informal vlog engagement statistically linked with identity relevant self-perception, and which learner capabilities govern whether platform affordances are actualized at all? This study addresses these questions by positioning two social affective experiences, social presence and learning engagement, as experiences statistically linking vlog learning with multilingual identity, and digital literacy as the capability that conditions those associations.

The focal practice, short video vlog assisted informal digital language learning, denotes learners’ self directed, interest driven engagement with target language and multilingual vlogs outside formal instruction, spanning the consumption of vlog content, interactive participation around it, and the appropriation of vlog encountered language for one’s own use (Frobenius, 2011; Sun et al., 2026). The focal criterion variable, self-perceived multilingual identity, refers here to learners’ current explicit self identification as multilingual, a holistic sense of self that transcends discrete single language identities and integrates their evaluations of, experiences with, and emotions toward multiple languages (Fisher et al., 2024; Shi and Xu, 2025). Conceptualized through this 3Es structure of evaluation, experience, and emotion, self-perceived multilingual identity is theoretically relevant to vlog environments, which simultaneously provide linguistic models, cultural representations, occasions for social visibility, and objects of affective response (Henry, 2017; Li and Ding, 2026; Shi and Xu, 2025).

The first experience, social presence, is the degree to which others are felt as real and proximate in a mediated environment, the sense of human contact, warmth, and immediacy that makes a digital space feel populated (Kreijns et al., 2022; Wei et al., 2012). Because short video platforms surround content with comments, danmu, likes, and visible communities, they are well suited to being associated with this felt co presence, which may align multilingual exposure with experiences of belonging and recognition (Ou et al., 2024; Yang et al., 2022). The second experience, learning engagement, is learners’ multidimensional investment in a learning activity, affective, cognitive, and linguistic, beyond the frequency or duration of that activity (Arndt, 2023; Hiver et al., 2024; Svalberg, 2009). Engagement corresponds to deeper processing of exposure: noticing language, interpreting culture, and relating both to the self (Arndt, 2023; Hiver et al., 2024). Theory and evidence further suggest that these experiences are ordered: perceived presence is associated with the willingness to invest, so that social presence and engagement form a social to cognitive sequence of associations (Gao et al., 2024; Lei et al., 2026; Wu, 2023).

Whether any of these affordances are perceived and actualized, however, depends on the learner. Digital literacy (rooted in the new media literacy tradition’s distinction between functional and critical, consuming and prosuming competencies) is the capacity to access, interpret, evaluate, and responsibly produce content in networked environments (Lee et al., 2015; Luan et al., 2023). In short video settings, this capacity is expected to condition whether learners can locate worthwhile content, read multimodal meaning, judge the reliability of linguistic and cultural representations, manage distraction, and turn fleeting input into usable resources (Li and Ding, 2026; Luan et al., 2023). Digital literacy is thus the agentic capability that the ecological account requires: a boundary condition expected to condition the associations from vlog learning to social presence, engagement, and identity for learners equipped to act on what platforms afford, and to weaken them for learners who are not (Guamos and Estremera, 2025; Li and Ding, 2026).

Despite these developments, three gaps remain in the literature. First, research on social media and language learning has generated substantial evidence on skills, motivation, interaction, and learning related variables, yet self-perceived multilingual identity has received comparatively little attention as a social affective and digitally mediated criterion variable in short video vlog based informal learning (Barrot, 2022; Guamos and Estremera, 2025; Shi and Xu, 2025; Sun et al., 2026). Second, existing vlog related studies have examined affordances, learner agency, and platform specific practices, yet how vlog assisted informal learning is statistically associated with identity relevant self-perception, that is, through which experiential associations and in what theoretically specified order, remains underspecified (Arndt, 2023; Gao et al., 2024; Li and Ding, 2026; Sun et al., 2026; Wu, 2023). Third, digital literacy has been developed and validated as an important construct for EFL learners in new media environments, yet its role as a boundary condition within a short video vlog based model of self-perceived multilingual identity has received limited empirical attention (Luan et al., 2023; Palacios-Hidalgo and Huertas-Abril, 2025).

These gaps, and the social concern that motivates them, converge on a single theoretical question with a pressing practical edge: through what experiences, and for which learners, is everyday informal exposure to multilingual short video content associated with who learners take themselves to be as multilingual speakers? The present study addresses this question in two parts:

- RQ1. To what extent is short video vlog assisted informal digital language learning associated with self-perceived multilingual identity through learners’ experiences of social presence and learning engagement?
- RQ2. To what extent does learners’ digital literacy condition these associations?

To address these questions, the study uses a cross sectional questionnaire with Chinese university language learners, combining adapted and validated instruments for each construct (Arndt, 2023; Gao et al., 2024; Luan et al., 2023; Shi and Xu, 2025; Sun et al., 2026). The hypothesized model corresponds to a conditional process specification (Model 85) in which social presence and learning engagement operate as serial intermediate constructs and digital literacy moderates the paths originating in vlog learning (Hayes, 2022); the same moderated serial indirect-association structure has recently been applied to digital social interaction and learning (Lei et al., 2026; Wang and Wang, 2025a). Because the data are cross sectional and self reported, the analysis warrants claims about association and conditional indirect pattern, not causation.

The study makes three contributions. Theoretically, it relocates vlog assisted informal learning from the familiar terrain of skills and motivation to the social affective terrain of identity, specifying self-perceived multilingual identity as the criterion variable examined in relation to everyday digital practice (Shi and Xu, 2025; Sun et al., 2026). Analytically, it brings together two literatures that rarely meet (online social presence and informal second language engagement) within a single theoretically ordered specification, examining how a socially populated platform is statistically associated with engagement and identity related self-perception (Arndt, 2023; Gao et al., 2024; Kreijns et al., 2022). Conditionally, it brings new media literacy into the model as the learner capability that conditions the extent to which platform affordances are actualized, giving empirical content to the ecological claim that affordances without agency are inert (Li and Ding, 2026; Luan et al., 2023). Practically, as multilingual resources increasingly reach learners through short video feeds, the findings can help educators judge when such consumption is likely to be accompanied by social connection, genuine engagement, and identity related self-perception, and design informal and semi formal learning spaces that are socially present, affectively supportive, and critically literate (Gao et al., 2024; Guamos and Estremera, 2025; Luan et al., 2023).

## 2 Literature review and hypothesis development

This chapter reviews the theoretical and empirical literature underpinning the study’s five focal constructs and develops the hypotheses to be tested. It is organised in four parts. Section 2.1 introduces short video vlog assisted informal digital language learning and links it to self-perceived multilingual identity. Section 2.2 develops social presence as a social affective association. Section 2.3 develops learning engagement as a language learning association and considers the two intermediate constructs in sequence. Section 2.4 introduces digital literacy as a boundary condition.

These constructs are not assembled *ad hoc* but follow from the ecological premise that an environment’s affordances become salient only as learners perceive and actualise them through agency (Jiang and Zhang, 2019; Xue and Churchill, 2019). That premise structures the model in three ways: the model focuses on experiences through which learners take up what the environment affords; it specifies a theoretically ordered social-to-cognitive sequence involving social presence and learning engagement; and it treats digital literacy as a boundary condition because affordances depend on learners’ capability to act on platform opportunities (Gao et al., 2024; Jiang and Zhang, 2019; Li and Ding, 2026; Tong et al., 2024).

### 2.1 Short video vlog assisted informal digital language learning and multilingual identity

Informal digital language learning has become an increasingly prominent strand of second language and multilingual development research, in large part because learners now access language resources through everyday digital activities such as social networking, online video viewing, gaming, music streaming, and mobile communication (Arndt, 2023; Lee, 2019; Lee and Dressman, 2018). The label informal digital learning of English (IDLE) has been used to describe self directed digital engagement with English beyond formal instruction (Lee, 2019; Lee and Dressman, 2018). Earlier research has linked informal digital language practices to vocabulary development, speaking, motivation, enjoyment, self efficacy, and confidence, while also noting considerable individual differences in how learners participate in, and benefit from, these practices (Arndt, 2023; Kuppens, 2010; Lai et al., 2015; Lee and Dressman, 2018).

Within this broader IDLE ecology, vlogs represent a tool specific and increasingly visible form of informal digital language learning (Dressman and Lee, 2021; Sun et al., 2026). A vlog is a video based form of blog that typically features personal narration, everyday experience, multimodal storytelling, and platform based interaction (Frobenius, 2011; Li and Ding, 2026; Yang et al., 2022). Sun et al. (2026) conceptualised vlog assisted informal language learning (VAILL) as a multidimensional form of learners’ informal engagement with vlogs and validated two instruments: VAILL U, for vlog use, and VAILL M, for vlog related motivation. The VAILL U scale distinguishes content consumption, interactive participation, and language application, thereby moving beyond the broad receptive productive distinction that has often dominated IDLE measurement (Lee and Lee, 2021; Sun et al., 2026; Zou et al., 2025). This distinction matters because watching a vlog, commenting on it, interacting with a vlogger, saving expressions, and applying vlog encountered language in one’s own communication represent qualitatively different forms of digital language practice (Sun et al., 2026).

Multilingual identity refers to learners’ explicit self identification as multilingual, grounded in an awareness of their linguistic repertoire and connected to a relatively stable yet developing sense of self across past, present, and future language experiences (Fisher et al., 2018; Haukås et al., 2021). Multilingualism itself can be understood as the capacity to engage functionally with more than one language in everyday life, regardless of uniform proficiency or identical learning histories across languages (Cenoz, 2013). Multilingual identity has been treated as a holistic construct that connects linguistic repertoire, beliefs, emotions, social contexts, educational trajectories, and possible selves (Forbes et al., 2021; Henry, 2017). Drawing on Fisher et al.’s (2024) 3Es framework of evaluation, experience, and emotion, Shi and Xu (2025) operationalised multilingual identity among Chinese university students, rendering the construct measurable as a multidimensional individual difference.

Beyond this individual-difference operationalisation, multilingual identity is also widely theorised as socially constructed and relational, negotiated and positioned through interaction rather than residing solely within the individual (Bucholtz and Hall, 2005; Darvin and Norton, 2015). On this view, learners claim, perform, and revise multilingual selves as they participate in linguistic communities, are recognised or not recognised by others, and move across languages in situated practice, so that identity is accomplished in interaction and shaped by relations of power and investment (Bucholtz and Hall, 2005; Darvin and Norton, 2015; Fisher et al., 2018). Digital multilingual environments extend this negotiation online: learners have been shown to construct a virtually translocal identity and to experiment with hybrid, fluid identifications across their repertoires as they communicate and translanguage in networked spaces (Ou et al., 2024; Palacios-Hidalgo and Huertas-Abril, 2025).

In this article, “multilingual identity” refers to the broader theoretical construct, whereas “self-perceived multilingual identity” refers to the survey-based criterion variable measured in the present study. The term “multilingual identity development” is reserved for the wider developmental literature and future longitudinal research, because the present cross-sectional design does not observe change over time.

The association between VAILL and self-perceived multilingual identity can be framed through several theoretical links. Vlogs provide authentic, multimodal, and culturally situated language input, which may correspond to learners’ language related experiences and evaluations of multilingualism (Dressman and Lee, 2021; Sun et al., 2026). Learners’ interactions with vloggers and other viewers may correspond to social experiences of belonging, affiliation, and recognition in multilingual digital spaces (Li and Ding, 2026; Yang et al., 2022). Exposure to different speakers, accents, lifestyles, and cultural scripts may further correspond to learners’ future oriented multilingual self positioning and emotional attachment to their multilingual repertoires (Henry, 2017; Shi and Xu, 2025). Studies of learner generated vlogs add that English vlogging involves identity construction, self presentation, social visibility, and imagined future selves (Li and Ding, 2026). Short video vlog assisted informal digital language learning is therefore expected to be positively associated with self-perceived multilingual identity.

*H1*: Short video vlog assisted informal digital language learning is positively associated with self-perceived multilingual identity.

### 2.2 Social presence

Social presence is commonly understood as the perceived presence of others in mediated communication, the extent to which users experience others as psychologically or socially “real” within a mediated environment (Kreijns et al., 2022; Tu, 2000). In online learning research, social presence has shifted from a static property of a medium to learners’ subjective perception of communication, immediacy, interpersonal connection, and the projection of real selves in mediated environments (Garrison, 2007; Wei et al., 2012; Wise et al., 2004). Wei et al. (2012) modelled social presence in online classrooms through co presence, intimacy, and immediacy, and Gao et al. (2024) drew on this three dimensional structure to examine social presence together with learning engagement in online learning. Comparable dynamics appear in synchronous video environments, where active participation is associated with heightened social presence and felt connection (Lin and Wang, 2026). Social presence is thus especially relevant to short video vlog learning, because vlog platforms are at once content delivery systems and socially populated environments, featuring comments, danmu, likes, shares, replies, follower networks, and algorithmically visible communities (Li and Ding, 2026; Yang et al., 2022).

Short video vlogs may be particularly relevant to social presence because they combine embodied narration, voice, facial expression, everyday scenes, viewer comments, danmu, and platform interaction (Sun et al., 2026; Yang et al., 2022). Research on danmu enabled recommendation vlogs shows that pseudo synchronicity, danmu content congruency, and parallelism correspond to viewers’ perceptions of co viewing, immediacy, and interpersonal warmth (Yang et al., 2022). Studies of online video based learning similarly highlight social presence as part of the wider community of inquiry ecology, in which mediated interaction is associated with learners’ sense of belonging and participation (Chen and Feng, 2023; Whiteside, 2015; Zydney et al., 2012). Wu (2023) also reported that informal digital learning of English was positively associated with social presence among Chinese EFL learners in online classes. Taken together, these findings suggest that learners who engage more actively with short video vlogs may report a stronger sense of being with others in a digital language learning space.

*H2*: Short video vlog assisted informal digital language learning is positively associated with social presence.

Social presence is also theoretically linked with self-perceived multilingual identity. Identity is widely understood as socially constructed, relational, and connected to the language mediated positioning of self and others (Bucholtz and Hall, 2005; Darvin and Norton, 2015; Ou et al., 2024). Multilingual identity is at once a private self label and a socially and affectively mediated form of belonging, recognition, and participation across linguistic communities (Fisher et al., 2018; Forbes et al., 2021; Shi and Xu, 2025). In digital multilingual environments, the perception that one is interacting with, observing, and being recognised by others may correspond to learners’ sense of legitimacy as multilingual participants (Ou et al., 2024; Palacios-Hidalgo and Huertas-Abril, 2025). Social presence can therefore serve as a socio affective condition under which vlog based multilingual exposure is experienced as socially meaningful, and as more than merely informational (Gao et al., 2024; Garrison, 2007; Kreijns et al., 2022). In this sense, learners’ perceived co presence, intimacy, and immediacy in short video environments are expected to be positively associated with self-perceived multilingual identity.

*H3*: Social presence is positively associated with self-perceived multilingual identity.

The preceding arguments also imply an indirect statistical association between short video vlog assisted informal digital language learning and self-perceived multilingual identity through social presence. VAILL involves content consumption, interactional participation, and language application, whereas social presence captures the degree to which learners experience such digital participation as socially populated, warm, immediate, and relational (Sun et al., 2026; Wei et al., 2012; Yang et al., 2022). Because self-perceived multilingual identity involves evaluation, experience, and emotion, the socially meaningful quality of vlog learning may represent an important indirect pattern associating digital media consumption with learners’ self positioning as multilinguals (Fisher et al., 2024; Shi and Xu, 2025). This study therefore expects a positive indirect statistical association through social presence.

*H4*: Social presence statistically represents the indirect positive association between short video vlog assisted informal digital language learning and self-perceived multilingual identity.

### 2.3 Learning engagement

Learning engagement is broadly defined as learners’ active participation, interest, and meaningful involvement in learning activities (Hiver et al., 2024). In educational psychology, engagement is often treated as context specific and multidimensional, capturing how learners act, think, feel, and interact in relation to a learning object or environment (Hiver et al., 2024; Oga-Baldwin, 2019). In second language acquisition, engagement has been conceptualised as a psycholinguistically meaningful construct involving behavioural participation, cognitive investment, emotional involvement, social interaction, and language focused attention (Hiver et al., 2024; Philp and Duchesne, 2016; Svalberg, 2009). Arndt (2023) argued that informal second language engagement should not be reduced to the frequency or duration of informal activities, and developed the ISLE questionnaire to capture affective, cognitive, and linguistic engagement in informal second language practices.

Short video vlog assisted informal digital language learning is expected to be associated with learning engagement because vlogs combine interest driven content, authentic input, multimodal resources, and platform interaction (Dressman and Lee, 2021; Sun et al., 2026). Vlog watching can involve affective engagement when learners enjoy or feel motivated by vlog content; cognitive engagement when they attend to meaning, compare perspectives, and interpret cultural information; and linguistic engagement when they notice words, phrases, pronunciation, subtitles, pragmatic routines, or cross linguistic differences (Arndt, 2023; Hiver et al., 2024). Prior informal learning research has linked digital language activities to motivation, self efficacy, enjoyment, vocabulary, speaking, and broader proficiency related variables (Arndt, 2023; Kuppens, 2010; Lai et al., 2015; Lee, 2019; Lee and Dressman, 2018). Studies of vlogs also indicate that vlog based learning corresponds to coherent oral production, intercultural interaction, motivation, digital literacy, creativity, and learner agency (Aldukhayel, 2021; Jin, 2024; Li and Ding, 2026). Thus, learners’ VAILL use is expected to be positively associated with learning engagement.

*H5*: Short video vlog assisted informal digital language learning is positively associated with learning engagement.

Learning engagement is also expected to be positively associated with self-perceived multilingual identity. Multilingual identity involves more than language knowledge; it encompasses learners’ participation in multilingual experiences, evaluations of their linguistic repertoire, and emotions attached to multilingual development (Fisher et al., 2018; Fisher et al., 2024; Shi and Xu, 2025). Affective engagement may correspond to enjoyment, pride, and emotional attachment to multilingual practices; cognitive engagement may correspond to reflection on language, culture, and self positioning; and linguistic engagement may correspond to attention to multilingual resources and their perceived usability in daily life (Arndt, 2023; Hiver et al., 2024; Shi and Xu, 2025). Research on self-perceived multilingual identity highlights the importance of language use habits, beliefs about multilingualism, imagined multilingual selves, open mindedness, and intercultural experiences (Forbes et al., 2021; Haukås et al., 2021; Henry, 2017). Engaged participation in short video vlog learning is therefore expected to correspond to stronger self-perceived multilingual identity.

*H6*: Learning engagement is positively associated with self-perceived multilingual identity.

Social presence and learning engagement are also expected to be positively associated. Community of inquiry research treats social presence as a key component of meaningful online learning experience, while online engagement research repeatedly shows that learners’ perceived interpersonal connection, belonging, and immediacy relate to stronger participation and investment in online learning (Garrison, 2007; Kreijns et al., 2022; Richardson and Swan, 2003; Wise et al., 2004). Empirical studies have found positive associations between social presence and online engagement across different learning contexts (Doo and Bonk, 2020; Gao et al., 2024; Miao et al., 2022; Molinillo et al., 2018; Wu, 2023). Gao et al. (2024) reported serial mediation involving social presence and learning engagement in online learning, and Wu (2023) found that social presence mediated the association between informal digital learning of English and online student engagement; comparable serial indirect patterns, in which relational and engagement related experiences are statistically ordered, have been modelled for digital social interaction in language and learning settings (Lei et al., 2026; Wang and Wang, 2025a). In short video vlog environments, perceived co viewing, interactional warmth, and response visibility may correspond to learners’ willingness to attend to content, participate, and invest affectively and linguistically in the learning process (Sun et al., 2026; Yang et al., 2022).

*H7*: Social presence is positively associated with learning engagement.

The above arguments suggest two indirect patterns involving learning engagement. First, short video vlog assisted informal digital language learning may be associated with self-perceived multilingual identity through learning engagement, since vlog based practices provide the content, interaction, and language resources with which learners engage affectively, cognitively, and linguistically (Arndt, 2023; Sun et al., 2026). Second, short video vlog assisted informal digital language learning may also be associated with self-perceived multilingual identity through the serial indirect pattern involving social presence and learning engagement, because socially meaningful vlog experiences may relate to deeper engagement, which in turn may correspond to self-perceived multilingual identity (Gao et al., 2024; Shi and Xu, 2025; Wu, 2023).

*H8*: Learning engagement statistically represents the indirect positive association between short video vlog assisted informal digital language learning and self-perceived multilingual identity.

*H9*: Short video vlog assisted informal digital language learning is positively associated with self-perceived multilingual identity through the serial indirect pattern involving social presence and learning engagement.

### 2.4 Digital literacy

Digital literacy refers to learners’ capacity to access, understand, evaluate, participate in, produce, distribute, and critically engage with digital content in networked environments (Lee et al., 2015; Luan et al., 2023). In EFL contexts, Luan et al. (2023) developed the English New Media Literacy scale by drawing on the functional and critical and the consuming and prosuming dimensions of new media literacy. Their framework includes functional consuming literacy, critical consuming literacy, functional prosuming literacy, and critical prosuming literacy, further operationalised through indicators such as consuming skill, understanding, analysis, synthesis, evaluation, prosuming skill, distribution, production, participation, and feedback (Lee et al., 2015; Luan et al., 2023). Although this study uses the term digital literacy, the construct is grounded in this new media literacy tradition, because short video vlog learning calls for access to content together with critical evaluation, multimodal interpretation, responsible interaction, and productive participation (Li and Ding, 2026; Luan et al., 2023). Treating a stable, learner level capability as a moderator of the associations involving digital social engagement is consistent with recent evidence that dispositional differences condition how far such engagement translates into affective and relational gains (Wang and Wang, 2025b).

Critically, this conception treats digital literacy as more than individual operational skill. It also includes learners’ capacity to read the sociotechnical environment in which media consumption occurs: algorithmic personalization filters and prioritizes what users encounter (Eg et al., 2023), platform infrastructures are shaped by commercial, financial, and regulatory logics rather than by educational purposes alone (Narayan, 2024), and algorithmic feeds may be interpreted as more or less responsive to users’ identities and goals (Taylor and Choi, 2022). In this sense, digital literacy is a critical capacity for evaluating platform curation, content credibility, commercial persuasion, and privacy risks, not simply a capacity to operate digital tools.

Digital literacy is expected to condition the association between short video vlog assisted informal digital language learning and self-perceived multilingual identity. Digital platforms provide linguistic, digital, social, and emotional affordances, but learners differ in whether they can identify high quality content, evaluate linguistic and cultural representations, use platform tools, manage distraction, interpret multimodal messages, and turn digital input into personally meaningful language resources (Li and Ding, 2026; Luan et al., 2023). Li and Ding (2026) found that the effective use of social media for learning depends on learners’ agentive use of digital tools, particularly in light of digital distraction and deficient digital ability. Guamos and Estremera (2025) similarly noted that social media based L2 learning requires digital literacy, privacy awareness, and careful instructional or learner guidance. Therefore, the positive association between VAILL and self-perceived multilingual identity is expected to be stronger among learners with higher digital literacy.

*H10*: Digital literacy moderates the positive association between short video vlog assisted informal digital language learning and self-perceived multilingual identity, such that the association is stronger when digital literacy is higher.

Digital literacy is also expected to condition the association between short video vlog assisted informal digital language learning and social presence. Learners with stronger digital literacy may be better able to use comments, danmu, hashtags, recommendation systems, subtitles, sharing functions, and community spaces to locate others, interpret interactional cues, and participate responsibly in online communication (Luan et al., 2023; Yang et al., 2022). Because social presence depends on perceived co presence, intimacy, immediacy, and interpersonal visibility, learners’ ability to navigate platform affordances may correspond to stronger perceptions of social connection in short video vlog spaces (Kreijns et al., 2022; Wei et al., 2012; Yang et al., 2022). In contrast, learners with lower digital literacy may consume vlog content more passively or experience platform interaction as fragmented, unreliable, or socially distant (Li and Ding, 2026; Luan et al., 2023).

*H11*: Digital literacy moderates the positive association between short video vlog assisted informal digital language learning and social presence, such that the association is stronger when digital literacy is higher.

Finally, digital literacy is expected to condition the association between short video vlog assisted informal digital language learning and learning engagement. Informal engagement requires more than exposure; it involves affective interest, cognitive investment, and attention to linguistic resources (Arndt, 2023). Learners with higher digital literacy may more effectively search for relevant vlog content, evaluate its reliability, use subtitles or translation tools, organise saved expressions, compare sources, and create their own learning artefacts from vlog input (Luan et al., 2023; Sun et al., 2026). These practices map closely onto the affective, cognitive, and linguistic dimensions of informal second language engagement (Arndt, 2023; Hiver et al., 2024). Conversely, learners with lower digital literacy may encounter more distraction, greater difficulty in evaluating content quality, or a weaker capacity to turn media exposure into language focused engagement (Guamos and Estremera, 2025; Li and Ding, 2026).

*H12*: Digital literacy moderates the positive association between short video vlog assisted informal digital language learning and learning engagement, such that the association is stronger when digital literacy is higher.

## 3 Methods

### 3.1 Research design

This study employed a cross sectional questionnaire design to examine the associations among short video vlog assisted informal digital language learning, social presence, learning engagement, digital literacy, and self-perceived multilingual identity among university language learners. The design positioned short video vlog assisted informal digital language learning as the independent variable (X), social presence (M1) and learning engagement (M2) as two serial intermediate variables, self-perceived multilingual identity as the criterion variable (Y), and digital literacy (W) as a moderator of the three associations originating from the independent variable. This conditional process structure corresponds to Model 85 (Hayes, 2022). Because the data were cross sectional and self reported, all estimates are interpreted as patterns of association and conditional indirect relations, and are not taken as evidence of causality.

### 3.2 Participants and procedure

The questionnaire was administered online to Chinese university students who had experience with short video platforms and foreign language learning. Eligible participants were enrolled at a university, had learned at least one foreign language, and had watched short video vlogs containing target language or multilingual content. Participation was voluntary and anonymous, and participants were informed of the research purpose, the voluntary nature of participation, and their right to withdraw before submission. After data screening (Section 3.3), the final analytic sample comprised *N* = 648 participants. The analytic sample had a mean age of 20.63 years (SD = 1.46, range = 18–25). Full sample characteristics are reported in Table 1. This study was approved (Approval No. CQUSJC2026012) by the Academic Ethics Committee, School of Journalism and Communication, Chongqing University, China, prior to the start of data collection.

**Table 1:** Sample characteristics.

Characteristic	Category	*n* (%)
Age (M, SD; range)		20.63 (1.46); 18–25
Gender	Male	229 (35.3%)
	Female	373 (57.6%)
	Non-binary/other	29 (4.5%)
	Prefer not to say	17 (2.6%)
Year level	First-year UG	157 (24.2%)
	Second-year UG	149 (23.0%)
	Third-year UG	127 (19.6%)
	Fourth-year UG	101 (15.6%)
	Master’s	76 (11.7%)
	Doctoral	24 (3.7%)
	Other	14 (2.2%)
Major	Language-related	182 (28.1%)
	Humanities/social science	136 (21.0%)
	STEM	139 (21.5%)
	Business/management	97 (15.0%)
	Arts/media	63 (9.7%)
	Other	31 (4.8%)
Foreign languages	One	407 (62.8%)
	Two or more	241 (37.2%)

UG, undergraduate. Percentages are computed within the valid analytic sample.

Participants engaged with short-video vlogs on major Chinese platforms, for example Douyin and Bilibili, environments characterized by dense comment and danmu interaction and by content spanning target-language instruction, multilingual lifestyle vlogging, and culturally situated narration (Li and Ding, 2026; Yang et al., 2022).

### 3.3 Data screening and missing data treatment

The raw dataset contained 675 responses to 61 focal scale items. Two issues required attention before analysis: careless responding and item level missingness. These were addressed in sequence.

**Figure 1:**
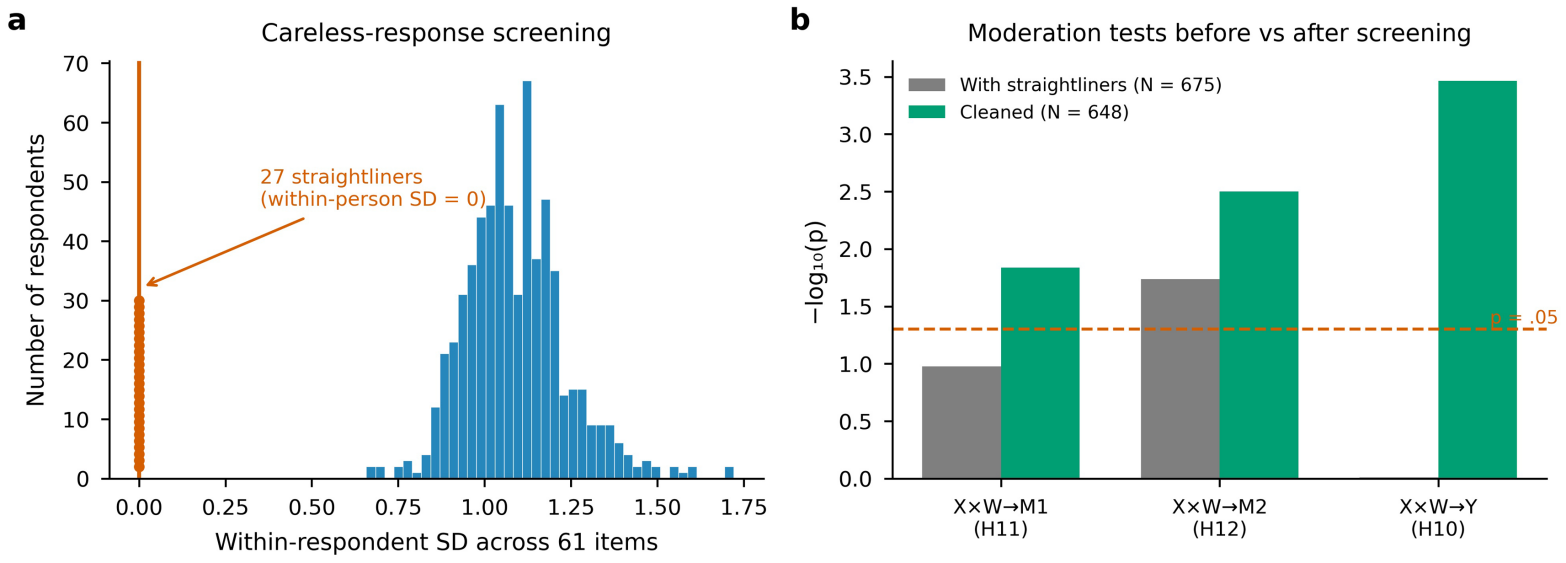
Careless response screening and its statistical implications. **(a)** Distribution of within respondent standard deviation across the 61 items; the 27 straightliners (SD = 0) are isolated from the rest of the sample. **(b)** Statistical evidence (−log₁₀ p) of the three X × W interactions before (*N* = 675) and after (*N* = 648) removing the careless records; the dashed line marks *p* = 0.05.

First, the data were screened for careless or insufficient effort responding using an intra individual response variability criterion (Dunn et al., 2018; Meade and Craig, 2012). For each respondent, the standard deviation of responses across all 61 items was computed; a value of zero indicates that the respondent selected an identical option for every item (complete straightlining), a longstring spanning the entire instrument. Twenty seven respondents (4.0%) met this criterion, each having entered the same response (all 1 s or all 7 s) across all 61 items, producing within respondent SD = 0 and a maximum longstring of 61. Because such records carry no usable covariance information and artificially inflate internal consistency and bivariate associations, all 27 were removed, yielding a valid sample of *N* = 648. As shown later (Section 4.6 and Figure 1), retaining these records materially distorted the moderation results.

Second, after removing careless responders, item level missingness among the 648 valid cases was low (590 missing values across 39,528 item responses, i.e., 648 cases × 61 items; 1.49%), with no item exceeding 2.62% missing. However, because the missing values were scattered, 390 of the 648 cases (60.2%) had at least one missing item, so listwise deletion would have discarded the majority of the sample and severely reduced statistical power. Consistent with best practice for low rates of randomly distributed missingness (Enders, 2010; Schafer and Graham, 2002), the missing values were therefore imputed at the item level using an expectation maximization based iterative (regression) imputer, with imputed values constrained to the 1–7 response range. All 648 cases were retained for every subsequent analysis. Three negatively worded items (M1_4R, M1_8R, and Y_15R) were reverse coded prior to scoring.

The pattern of missingness was consistent with data missing completely at random: Little’s MCAR test was non-significant, χ^2^(12221) = 12278.40, *p* = 0.355, and a logistic regression predicting whether a case had any missing value from observed background and study variables was likewise non-significant (likelihood-ratio *p* = 0.19), indicating no detectable association between missingness and observed covariates. The substantive results were unchanged in a complete-case re-analysis of the 258 fully observed cases (Table 2).

**Table 2:** Robustness and sensitivity analyses.

Specification	Direct c′	Total indirect	Serial indirect	X × W interactions
Serial indirect model – no controls	0.171	0.337	0.077	**M1 0.019 / M2 0.003 / Y < 0.001**
Serial indirect model – main controls	0.177	0.340	0.076	**M1 0.015 / M2 0.003 / Y < 0.001**
Serial indirect model – expanded controls	0.175	0.340	0.074	**M1 0.016 / M2 0.004 / Y < 0.001**
SEM (ML estimator)	0.177	—	—	**all paths *p* < 0.001**
Complete-case (listwise, *n* = 258)	0.221	0.307	0.111	Pattern retained
One language (*n* = 407)	0.183	0.338	0.077	Pattern retained
Two+ languages (*n* = 241)	0.169	0.342	0.072	Pattern retained
Serial indirect model – background-expanded controls	0.169	0.347	0.076	M1 0.012 / M2 0.004 / Y < 0.001

Estimates are unstandardized. “Expanded controls” adds age, gender, and year level to the three main controls; “background-expanded controls” instead adds socioeconomic status, dialect background, overseas experience, and academic major. For the control-set rows, the final column reports the *p* values of the three X × W interactions (on M1, M2, and Y). SEM, structural equation modeling; ML, maximum likelihood.

### 3.4 Measures

Unless otherwise noted, all focal constructs were measured on seven point Likert scales. Short video vlog assisted informal digital language learning was rated for frequency (1 = never to 7 = always); social presence, learning engagement, self-perceived multilingual identity, and digital literacy were rated for agreement (1 = strongly disagree to 7 = strongly agree). Higher scores indicate higher levels of each construct. Full bilingual item wording is provided in Supplementary material. Internal consistency estimates reported below are for the valid analytic sample (*N* = 648).

All adapted instruments were first translated from English into Chinese and then independently back-translated into English. Discrepancies between the original and back-translated versions were reviewed and resolved through discussion. The contextual adaptation of items to short-video vlog-assisted informal language learning was further reviewed by experts in applied linguistics and digital language learning to evaluate clarity, content relevance, and contextual appropriateness.

Measured with an adapted 13 item version of the VAILL Use scale (Sun et al., 2026), spanning three factors: content consumption, interactive participation, and language application. Sample items include “I watch short video vlogs that use the target language or multiple languages” (content consumption) and “I try to use expressions learned from short video vlogs in everyday communication” (language application). Cronbach’s *α* = 0.958. Full wording appears in Supplementary material.

Measured with an adapted 12 item version of the Social Presence scale (Wei et al., 2012); used in online learning research by Gao et al. (2024), spanning three factors, co presence, intimacy, and immediacy, contextualized to short video vlog environments. Sample items include “When learning languages through short video vlogs, I feel that I am not learning alone” (co presence) and an immediacy item indicating that comments, danmu, and vlogger replies make the interaction feel immediate. Two items are reverse coded. Cronbach’s α = 0.950. Full wording appears in Supplementary material.

Measured with an adapted 9 item version of the Informal Second Language Engagement questionnaire (Arndt, 2023), comprising three factors, affective, cognitive, and linguistic engagement. The behavioural engagement factor of the original instrument was excluded to avoid conceptual overlap with the independent variable. Sample items include “I find it interesting to learn the target language or multiple languages through short video vlogs” (affective) and “I pay special attention to vocabulary, phrases, subtitles, pronunciation, or sentence patterns” (linguistic). Cronbach’s α = 0.933. Full wording appears in Supplementary material.

Measured with an adapted 15 item version of Shi and Xu’s (2025) self-perceived multilingual identity scale, grounded in the 3Es framework and spanning three factors: evaluation, experience, and emotion, contextualized to informal digital learning. Sample items include “I regard being able to understand or use multiple languages as part of who I am” (evaluation) and “Learning or using multiple languages makes me feel happy” (emotion). One item is reverse coded. Cronbach’s α = 0.955. Full wording appears in Supplementary material.

Measured with an adapted 12 item version of the English New Media Literacy scale (Luan et al., 2023), spanning four factors: digital access and operation, multimodal understanding, critical evaluation, and participation and creation. Sample items include “I can identify possible stereotypes, biases, exaggerations, or algorithmic persuasion in short video vlogs” (critical evaluation) and “I can understand how text, speech, subtitles, images, and background music jointly convey meaning in short video vlogs” (multimodal understanding). Cronbach’s α = 0.955. Full wording appears in Supplementary material.

Three control variables were included: self rated target language proficiency (1 = very low to 7 = very high), the number of foreign languages learned (excluding mother tongue and dialects), and general daily short video platform use intensity (1 = less than 30 min to 5 = more than 3 h).

### 3.5 Analytic strategy

Analyses proceeded in five stages. First, descriptive statistics and bivariate correlations were computed. Second, the measurement model was evaluated: internal consistency via Cronbach’s α, McDonald’s *ω*, and composite reliability (CR); convergent validity via average variance extracted (AVE) and standardized loadings from a confirmatory factor analysis (CFA); and discriminant validity via the Fornell–Larcker criterion and the heterotrait monotrait ratio (HTMT) (Fornell and Larcker, 1981; Henseler et al., 2015). Model fit was assessed with χ^2^/df, CFI, TLI, RMSEA, and SRMR. Third, common method bias was examined using Harman’s single factor test and full collinearity variance inflation factors (VIFs; Kock, 2015). Fourth, the hypotheses were tested: a serial indirect-association model, structurally equivalent to Hayes’s (2022) Model 6, provided estimates for the direct, specific indirect, and serial indirect associations (H1–H9), and the full conditional process model, structurally equivalent to Model 85, provided the interaction and conditional indirect associations, together with the index of moderated mediation, for H10–H12. The paths were estimated by ordinary least squares regression in Python; the PROCESS macro itself was not used. All continuous predictors were mean centred before forming product terms, and percentile bootstrap confidence intervals based on 5,000 resamples were used for all indirect and conditional indirect associations. Fifth, robustness and sensitivity analyses examined alternative control variable sets, an alternative maximum likelihood structural equation estimator, a complete case (listwise) re estimation, and subgroup analyses by language repertoire breadth. Analyses were conducted in Python using statsmodels and semopy.

## 4 Results

### 4.1 Descriptive statistics and correlations

Means, standard deviations, reliabilities, and bivariate correlations are reported in Table 3. The five focal constructs were all positively and significantly intercorrelated (rs = 0.518–0.673, ps < 0.001). The independent variable correlated most strongly with self-perceived multilingual identity (*r* = 0.562), and the two mediators correlated substantially with the criterion variable (social presence *r* = 0.662; learning engagement *r* = 0.673). Digital literacy correlated moderately with all constructs (rs = 0.248–0.514).

**Table 3:** Descriptive statistics, reliabilities, and correlations.

Variable	M	SD	1	2	3	4	5	6	7	8
1. X	4.06	1.04	**0.82**							
2. M1	3.98	0.99	0.518***	**0.80**						
3. M2	3.99	1.01	0.552***	0.626***	**0.81**					
4. Y	4.31	0.94	0.562***	0.662***	0.673***	**0.78**				
5. W	4.12	1.06	0.248***	0.514***	0.470***	0.493***	**0.82**			
6. Proficiency	3.50	1.28	0.013	0.069	0.087*	0.132**	0.081*	—		
7. Languages	1.48	0.69	−0.016	−0.056	−0.008	0.000	−0.027	−0.002	—	
8. Daily use	3.04	1.02	0.300***	0.119**	0.118**	0.136***	−0.051	0.024	0.021	—

*N* = 648. Square roots of the average variance extracted (AVE) for the five latent constructs are on the diagonal (in bold positions). X = vlog assisted informal digital language learning; M1 = social presence; M2 = learning engagement; Y = self-perceived multilingual identity; W = digital literacy. *p < 0.05, **p < 0.01, ***p < 0.001.

### 4.2 Reliability, convergent validity, and the measurement model

All scales demonstrated excellent internal consistency and convergent validity (Table 4). Cronbach’s α ranged from 0.933 to 0.958, McDonald’s ω from 0.944 to 0.963, and CR from 0.944 to 0.963. AVE ranged from 0.615 to 0.670, exceeding the 0.50 threshold for every construct.

**Table 4:** Reliability and convergent validity.

Construct	Items	α	ω	CR	AVE
Vlog-assisted informal learning (X)	13	0.958	0.963	0.963	0.665
Social presence (M1)	12	0.950	0.956	0.956	0.644
Learning engagement (M2)	9	0.933	0.944	0.944	0.653
Self-perceived multilingual identity (Y)	15	0.955	0.960	0.960	0.615
Digital literacy (W)	12	0.955	0.961	0.961	0.670

α, Cronbach’s alpha; ω, McDonald’s omega; CR, composite reliability; AVE, average variance extracted.

**Figure 2:**
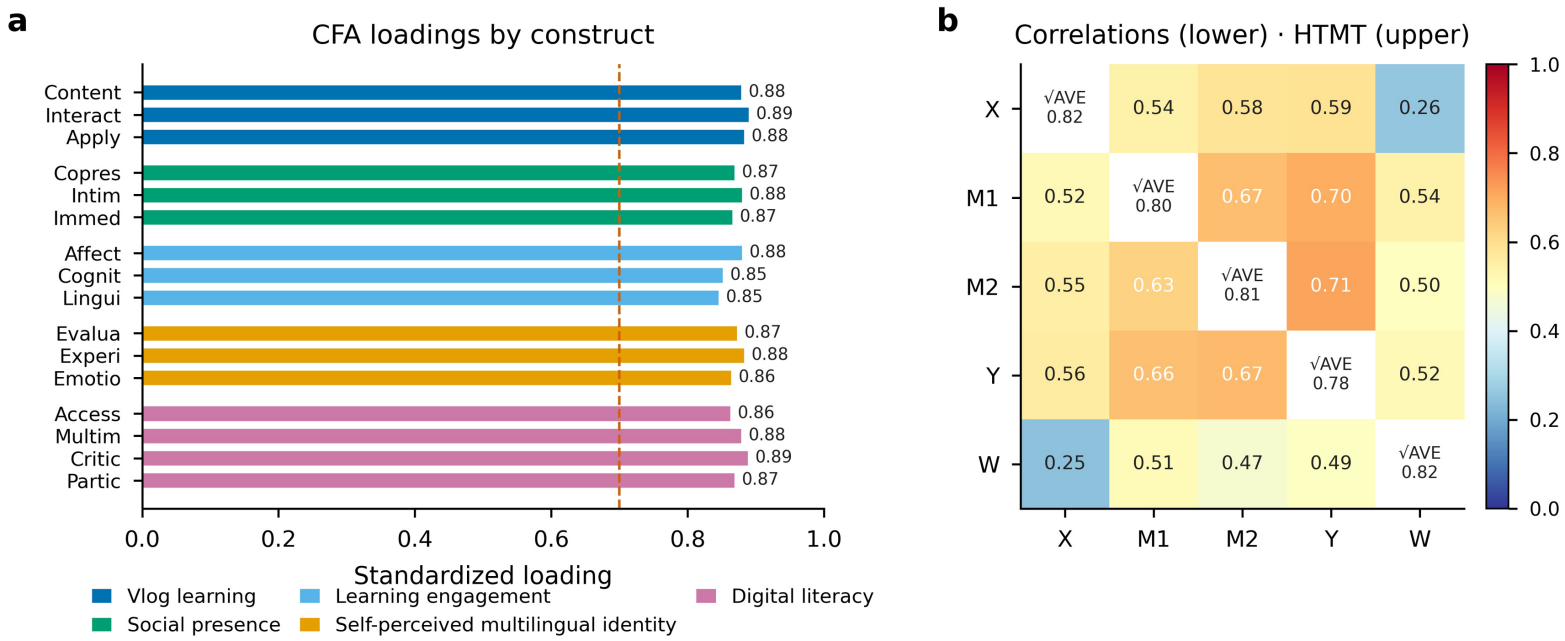
Measurement model evidence. **(a)** Standardized CFA loadings for the 16 dimension level parcels, grouped by construct (dashed line = 0.70 threshold). **(b)** Inter construct correlations (lower triangle) and HTMT ratios (upper triangle); √AVE values appear on the diagonal.

A correlated five factor CFA was estimated using the 16 theoretically specified dimension level parcels as indicators. The model fit the data well, χ^2^(94) = 110.29, *p* = 0.120, CFI = 0.998, TLI = 0.998, RMSEA = 0.016, SRMR = 0.016 (Table 5; Figure 2). All standardized loadings were high and significant (0.85–0.89, ps < 0.001).

**Table 5:** Confirmatory factor analysis fit indices.

Index	Value	Criterion
χ^2^ (df)	110.29 (94)	—
χ^2^/df	1.17	<3
*p* value	0.120	>0.05
CFI	0.998	≥0.95
TLI	0.998	≥0.95
RMSEA	0.016	≤0.06
SRMR	0.016	≤0.08
Std. loadings	0.85–0.89	≥0.50

Five factor model with 16 dimension level parcels (*N* = 648).

The 16 parcels were constructed transparently as unweighted means of the *a priori* first-order sub-dimensions rather than to maximize fit. Because parcelling can mask item-level misfit, a full item-level five-factor CFA with all 61 indicators was additionally estimated and is reported in Supplementary material (Section S8); it fit acceptably, indicating that the parcel-level solution is corroborated by item-level evidence.

Discriminant validity was supported by both criteria (Table 6). For every construct, the square root of AVE exceeded its correlations with all other constructs (Fornell–Larcker), and all HTMT ratios were well below the conservative 0.85 threshold (maximum HTMT = 0.712).

**Table 6:** Discriminant validity: heterotrait monotrait ratios (HTMT).

Variables	X	M1	M2	Y	W
X	—				
M1	0.544	—			
M2	0.584	0.665	—		
Y	0.588	0.696	0.712	—	
W	0.260	0.541	0.499	0.517	—

All HTMT ratios are below 0.85. See Table 3 for the Fornell–Larcker comparison (√AVE on the diagonal).

To test the two most closely related construct boundaries directly, nested confirmatory models were compared. Collapsing social presence and learning engagement into a single factor produced significantly worse fit than the five-factor solution (four-factor CFI = 0.932 vs. five-factor CFI = 0.998; Δχ^2^(4) = 567.2, *p* < 0.001). At the item level, a model forcing the three language application items and the three linguistic engagement items onto one factor fit far worse than a two-factor model separating them (one-factor CFI = 0.694 vs. two-factor CFI = 1.000; Δχ^2^(1) = 650.8, *p* < 0.001), with the two factors correlated but distinct (*r* = 0.52).

### 4.3 Common method bias

Three diagnostics were used to assess common method variance, while recognizing that none can establish its absence. Harman’s single factor test showed that the first unrotated factor accounted for 40.80% of the variance, below the 50% threshold. Full collinearity VIFs ranged from 1.50–2.42, well below the 3.3 criterion (Kock, 2015), indicating no evidence of pathological collinearity or a dominant method factor.

Because Harman’s test and collinearity indices are relatively insensitive, a confirmatory single-factor model was additionally estimated, in which all 16 parcels were forced onto one common factor; it fit the data poorly (CFI = 0.626, TLI = 0.568, RMSEA = 0.217), far worse than the five-factor measurement model (CFI = 0.998). These diagnostics suggest that common method variance is unlikely to account for the findings, but they do not rule it out because all focal constructs were self-reported at one time point.

### 4.4 Direct, indirect, and serial associations

**Figure 3:**
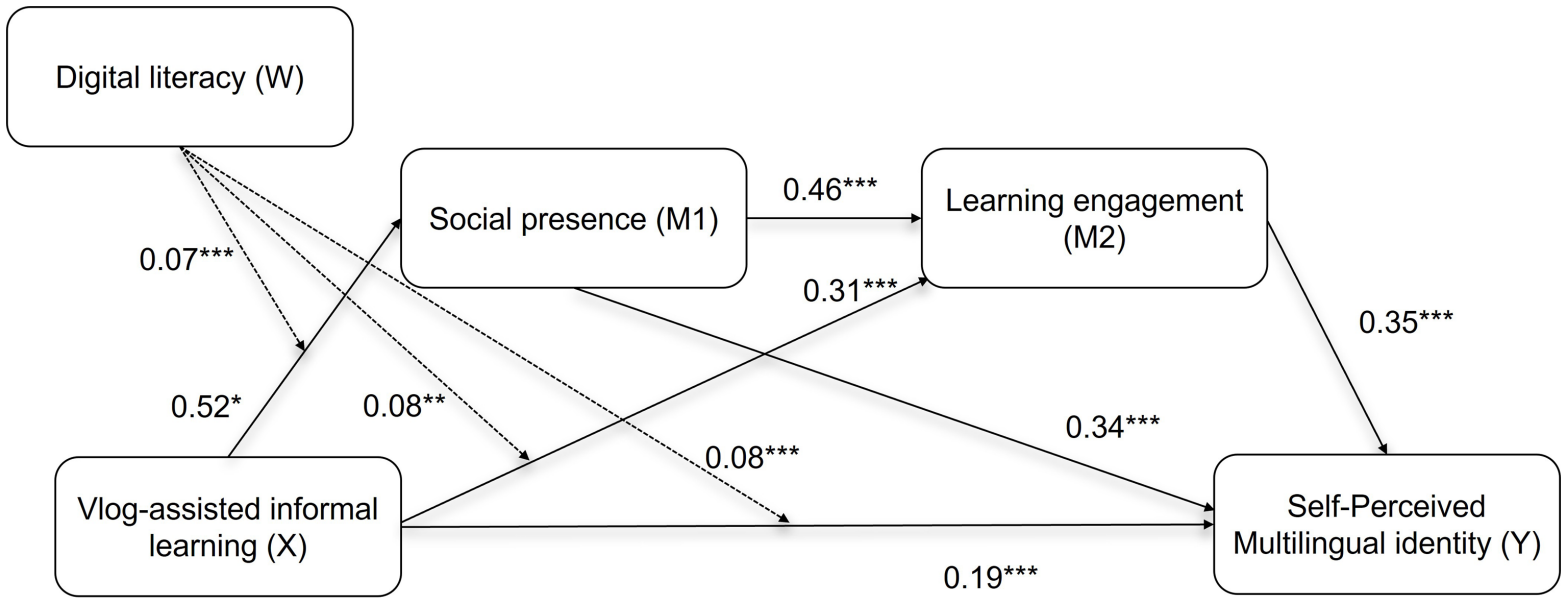
Estimated statistical associations. The estimated conditional process model (standardized coefficients). Solid arrows = direct and indirect statistical associations; dashed orange arrows = moderation of the three X associations by digital literacy (W). ***p < 0.001, **p < 0.01, *p < 0.05.

The serial indirect-association model (controlling for proficiency, number of languages, and daily use) explained substantial variance in all endogenous variables (R^2^ = 0.276 for social presence, 0.467 for learning engagement, and 0.578 for self-perceived multilingual identity). Standardized path coefficients are displayed in Figure 3. Vlog assisted informal learning was associated with social presence (*β* = 0.52) and learning engagement (*β* = 0.31); social presence was associated with learning engagement (*β* = 0.46); and both intermediate variables were associated with self-perceived multilingual identity (social presence *β* = 0.34; learning engagement *β* = 0.35), all ps < 0.001. The direct association of vlog learning on self-perceived multilingual identity remained significant (*β* = 0.19, *p* < 0.001), indicating a partial indirect pattern. These results support H1, H2, H3, H5, H6, and H7.

**Figure 4:**
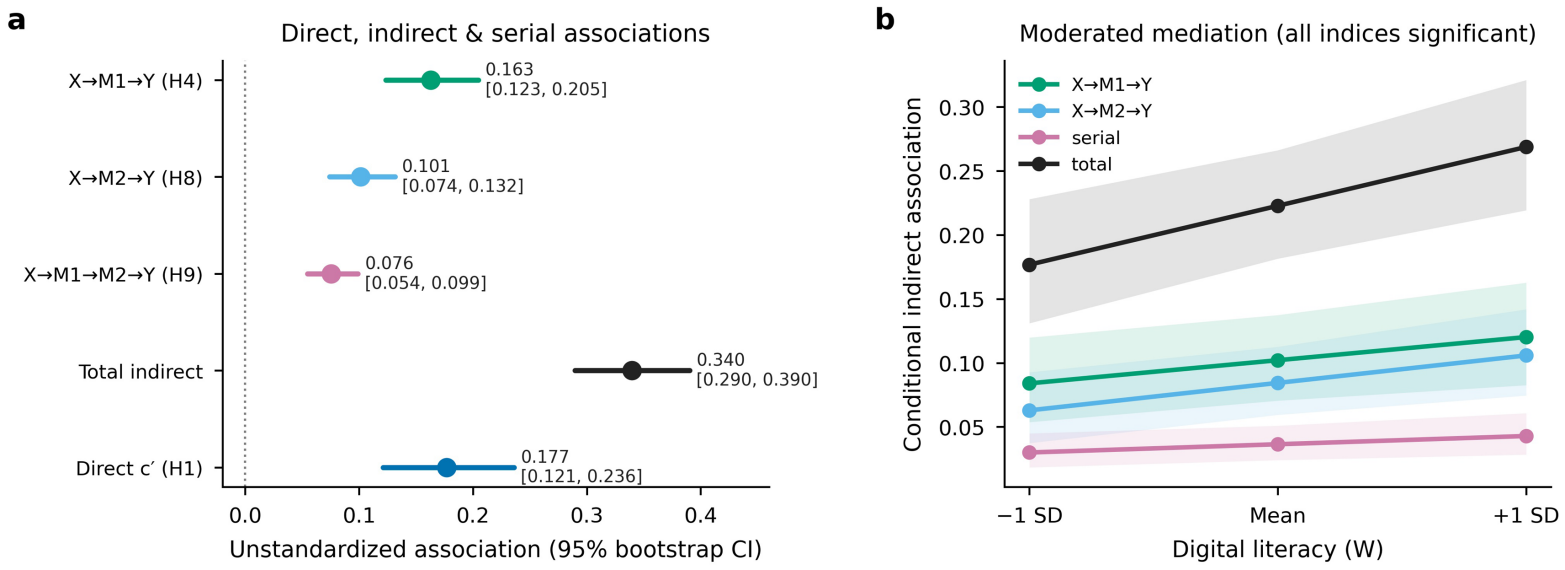
Indirect statistical associations. **(a)** Forest plot of the specific indirect, serial, total indirect, and direct associations with 95% bootstrap confidence intervals. **(b)** Conditional indirect associations across levels of digital literacy (W); all indices of moderated mediation were significant.

Bootstrap tests indicated that all three indirect associations were statistically different from zero (Table 7; Figure 4a). The specific indirect association through social presence was significant (estimate = 0.163, 95% CI [0.123, 0.205]; H4), as was the indirect association through learning engagement (estimate = 0.101, 95% CI [0.074, 0.132]; H8) and the serial indirect association through social presence and then learning engagement (estimate = 0.076, 95% CI [0.054, 0.099]; H9). The total indirect association was 0.340 (95% CI [0.290, 0.390]) and the total association was 0.517 (95% CI [0.456, 0.577]). Thus H4, H8, and H9 were supported.

**Table 7:** Direct, indirect, and serial associations.

Association	Estimate	95% bootstrap CI
X → M1 → Y (H4)	0.163	[0.123, 0.205]
X → M2 → Y (H8)	0.101	[0.074, 0.132]
X → M1 → M2 → Y (H9)	0.076	[0.054, 0.099]
Total indirect	0.340	[0.290, 0.390]
Direct association c′ (H1)	0.177	[0.121, 0.236]
Total association	0.517	[0.456, 0.577]

Unstandardized estimates; 5,000 bootstrap resamples. Controls: proficiency, number of languages, daily use.

### 4.5 Moderated indirect associations

**Figure 5:**
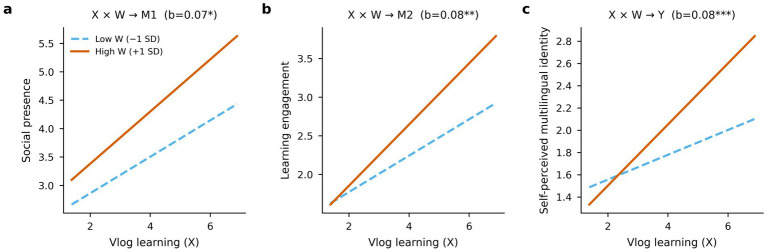
Simple slope plots. Simple slope plots of the moderation of digital literacy (W) on the three paths from vlog learning (X) to **(a)** social presence, **(b)** learning engagement, and **(c)** self-perceived multilingual identity. In every panel the slope is steeper at high W (+1 SD) than at low W (−1 SD).

The full conditional process model (Table 8) showed that digital literacy significantly moderated all three paths originating from vlog learning. The X × W interaction was significant for social presence (*b* = 0.066, *p* = 0.015; H11), for learning engagement (*b* = 0.076, *p* = 0.003; H12), and for self-perceived multilingual identity (*b* = 0.077, *p* < 0.001; H10). In every case the association between vlog learning and the criterion variable was stronger at higher levels of digital literacy, as illustrated by the simple slope plots in Figure 5. Therefore H10, H11, and H12 were all supported.

**Table 8:** Regression estimates for the conditional process model.

Outcome	Path	b	SE	*t*	*p*
M1	X → M1	0.390	0.031	12.58	<0.001***
M1	W → M1	0.381	0.029	13.04	<0.001***
M1	X × W → M1	0.066	0.027	2.45	0.015*
M2	X → M2	0.316	0.033	9.54	<0.001***
M2	M1 → M2	0.349	0.038	9.25	<0.001***
M2	W → M2	0.196	0.031	6.23	<0.001***
M2	X × W → M2	0.076	0.026	2.96	0.003**
Y	X → Y (c′)	0.193	0.029	6.57	<0.001***
Y	M1 → Y	0.261	0.033	7.84	<0.001***
Y	M2 → Y	0.267	0.033	8.14	<0.001***
Y	W → Y	0.134	0.027	4.98	<0.001***
Y	X × W → Y	0.077	0.021	3.60	<0.001***

*R*^2^ = 0.435 (M1), 0.504 (M2), 0.601 (Y). Continuous predictors were mean centred. Controls were included but are omitted from the table. X = vlog learning; M1 = social presence; M2 = learning engagement; Y = self-perceived multilingual identity; W = digital literacy.

Because statistical significance is readily attained at this sample size, the practical magnitude of each interaction was also examined. The increment to explained variance from adding the X × W term was limited in absolute size: ΔR^2^ = 0.005 for social presence, 0.007 for learning engagement, and 0.008 for self-perceived multilingual identity. Translated into simple slopes, the association between vlog learning and self-perceived multilingual identity rose from b = 0.11 at low digital literacy (−1 SD) to *b* = 0.27 at high digital literacy (+1 SD); the corresponding slopes ran from 0.32 to 0.46 for social presence and from 0.24 to 0.40 for learning engagement.

Conditional indirect statistical associations and indices of moderated mediation are reported in Table 9; Figure 4b. Every indirect association was significant at low, mean, and high levels of digital literacy and increased monotonically with it; for example, the total indirect association rose from 0.177 at low digital literacy to 0.269 at high digital literacy. Critically, the index of moderated mediation was significant for the indirect association through social presence (95% CI [0.003, 0.033]), through learning engagement (95% CI [0.007, 0.035]), and through the serial indirect association (95% CI [0.001, 0.012]), as well as for the total indirect association (95% CI [0.020, 0.069]), indicating that the indirect associations varied by digital literacy.

**Table 9:** Conditional indirect statistical associations and index of moderated mediation.

Indirect association	Low W	Mean W	High W	Index (95% CI)
X → M1 → Y	0.084 [0.054, 0.120]	0.102 [0.070, 0.137]	0.120 [0.083, 0.163]	[0.003, 0.033]
X → M2 → Y	0.063 [0.037, 0.093]	0.084 [0.059, 0.113]	0.106 [0.074, 0.142]	[0.007, 0.035]
X → M1 → M2 → Y	0.030 [0.018, 0.045]	0.036 [0.024, 0.051]	0.043 [0.028, 0.061]	[0.001, 0.012]
Total indirect	0.177 [0.131, 0.228]	0.223 [0.181, 0.266]	0.269 [0.219, 0.321]	[0.020, 0.069]

Unstandardized conditional indirect associations with 95% bootstrap CIs in brackets; low and high W = ±1 SD (SD = 1.06). A 95% CI for the index that excludes zero indicates significant moderated mediation.

### 4.6 Robustness and sensitivity analyses

The substantive pattern was robust across specifications (Table 2). The direct, indirect, and serial associations were stable across models with no controls, the main controls, and an expanded set of demographic controls, and all three interactions remained significant throughout. An alternative maximum likelihood structural equation estimator reproduced the path coefficients, and a complete case (listwise) re estimation on the 258 fully observed cases preserved the pattern, indicating that the findings were not an artefact of the imputation.

To address possible omitted-variable bias from background characteristics, the models were additionally re-estimated with a control set that added socioeconomic status, dialect background, overseas experience, and academic major to the three main controls. The substantive pattern was unchanged: the direct association was 0.169, the total indirect association was 0.347, the serial indirect association was 0.076, and all three X × W interactions remained significant (on social presence *p* = 0.012, on learning engagement *p* = 0.004, and on self-perceived multilingual identity *p* < 0.001).

To address the possibility of alternative temporal orderings under a cross-sectional design, two reverse-order sensitivity analyses were conducted (Supplementary material, Section S14). First, self-perceived multilingual identity was specified as a predictor of vlog-assisted informal learning, social presence, and learning engagement; each association was positive and significant (standardized βs = 0.54, 0.53, and 0.57, all *p* < 0.001), indicating that a reversed ordering is statistically plausible. Second, a fully reversed conditional process model was estimated, placing self-perceived multilingual identity as the predictor and vlog-assisted informal learning as the criterion variable while retaining social presence and learning engagement as serial intermediate variables and digital literacy as the moderator; the direct and serial indirect associations remained significant (direct = 0.31; serial indirect = 0.043, 95% CI [0.027, 0.062]; total indirect = 0.275, 95% CI [0.212, 0.340]). These analyses were used only to gauge the plausibility of alternative orderings, not to identify causal direction. Because the simple and serial associations are essentially symmetric across specifications, the proposed model is interpreted as theoretically specified and associational rather than temporally demonstrated. One asymmetry was also observed: the moderated indirect associations were significant only in the hypothesized direction; given the small magnitude of the interactions, this asymmetry is treated as suggestive rather than decisive.

A final analysis underscores why careful data screening mattered. When the 27 careless straightliners were retained (the larger *N* = 675 sample), the interaction of vlog learning and digital literacy was non significant for social presence (*p* = 0.105) and for multilingual identity (*p* = 0.982), and significant only for learning engagement, a pattern that would have yielded nonsupport for H10 and H11. After the careless records were removed, all three interactions were significant (Figure 1). The invalid records, which lay on the main association diagonal with zero within person variance, were associated with nonsignificance for two of the three moderation terms; their removal was therefore a substantive data-quality decision rather than a cosmetic one.

### 4.7 Subgroup analyses by language repertoire breadth

Because the criterion variable concerns self-perceived multilingual identity, the model was re estimated separately for learners of one foreign language (*n* = 407) and learners of two or more (*n* = 241). The pattern was comparable across the two groups: the total indirect association was 0.338 in the one-language group and 0.342 in the two-or-more-languages group, with the serial indirect association significant in both. The proposed pattern was therefore comparable across learners differing in language repertoire breadth.

## 5 Discussion

This study examined a conditional process model in which short video vlog assisted informal digital language learning was associated with self-perceived multilingual identity among 648 university language learners, with social presence and learning engagement as serial intermediate variables and digital literacy as a moderator. The findings speak directly to the study’s two research questions. With respect to RQ1, which asked to what extent vlog assisted informal learning is associated with self-perceived multilingual identity through social presence and learning engagement, several patterns emerged. First, vlog assisted informal learning was positively associated with self-perceived multilingual identity, both directly and indirectly, with the direct association remaining significant alongside the indirect ones. Second, the data were consistent with an ordered, social to cognitive sequence of associations: vlog learning was associated with social presence, social presence was associated with learning engagement, and both were associated with self-perceived multilingual identity, so that the serial indirect association involving social presence and learning engagement was significant. With respect to RQ2, which asked to what extent digital literacy conditions these associations, digital literacy moderated all three associations originating in vlog learning; the associations of vlog learning with social presence, with learning engagement, and with self-perceived multilingual identity were each stronger at higher levels of digital literacy, and every index of moderated mediation was significant. In sum, all 12 hypotheses were supported.

These findings should be interpreted as evidence of theoretically specified associations rather than temporal or causal ordering. Although the model positions vlog-assisted informal learning before social presence, learning engagement, and self-perceived multilingual identity, the reverse ordering is also plausible: learners with stronger self-perceived multilingual identity may be more likely to seek multilingual vlog content, perceive platform interaction as socially meaningful, and report stronger engagement. The cross-sectional design cannot adjudicate between these interpretations.

The associations observed here align with, and extend, several strands of prior work. The model was framed by an ecological view of language learning, in which a digital environment is understood as a field of affordances, representing action potentials and opportunities for educational activities that emerge from the dynamic interaction between learners and their environment, which can be facilitative or inhibitory depending on what the learner perceives and acts upon (Jiang and Zhang, 2019; Xue and Churchill, 2019). In this tradition, “affordance and agency are mutually dependent” (Jiang and Zhang, 2019; Li and Ding, 2026): affordances become salient only as learners perceive and act on them, and the framework of “learner-actualized affordances” describes “how learners perceive, use, or even shape affordances” (Li and Ding, 2026; Tong et al., 2024). The present finding that the associations between vlog learning and its social affective correlates were conditional on digital literacy is consistent with this account.

The study also connects to research on informal digital language learning. Work in this tradition has documented associations between out of class digital activity and a range of language learning-related variables (Lee, 2019; Lee and Dressman, 2018), much of it situated in what Sauro and Zourou (2019) call the digital wilds, where “learning [that] does not take place within a digital context or community with a primary goal of language teaching and learning” is nonetheless rich. Reviews of social media language learning have catalogued affordances “including interactivity, collaboration, and authenticity” (Guamos and Estremera, 2025). The present focus on vlogs follows Sun et al. (2026), whose framework moves “beyond the broad receptive productive dichotomy to offer a more nuanced, tool specific framework for understanding learner engagement in VAILL.” Where much vlog and informal learning research has emphasized skills, motivation, and communication (Lee and Dressman, 2018; Sun et al., 2026), the present study relocates attention to a social affective and identity related correlate of such practice.

The role of social presence is consistent with the community of inquiry and social presence traditions. Kreijns et al. (2022) noted that social presence is originally defined as the “degree of salience of the other person in the interaction and the consequent salience of the interpersonal relationship,” and subsequent work has treated it as a multidimensional perception central to online experience (Garrison, 2007; Wei et al., 2012). The positive association between social presence and learning engagement parallels findings from formal online learning research (Gao et al., 2024; Wu, 2023), now observed in an informal short video context. The treatment of engagement, in turn, follows multidimensional conceptions. Engagement has been described as learners’ active participation, interest, and meaningful involvement in learning activities (Hiver et al., 2024) and as a construct “characterized by the investment of time, effort, and interest in learning activities” (Gao et al., 2024; see also Svalberg, 2009). The present distinction among affective, cognitive, and linguistic facets follows Arndt (2023), for whom linguistic engagement “refers to the part of this attention that is devoted to consciously noticing, decoding, and learning specific language features.”

Finally, the criterion variable connects to self-perceived multilingual identity scholarship. Following Bucholtz and Hall (2005, p. 586), identity can be understood as “the social positioning of self and other,” and Shi and Xu (2025) define self-perceived multilingual identity as individuals’ “explicit perceptions of themselves as multilingual, reflecting a holistic sense of self that transcends discrete language identities,” operationalized through the evaluation, experience, and emotion dimensions of the 3Es framework (Fisher et al., 2024). The observed association between digitally mediated practice and self-perceived multilingual identity is consistent with qualitative accounts in which learners construct “a virtually translocal identity” online (Ou et al., 2024) and “experiment with hybrid identities” across their linguistic repertoires (Palacios-Hidalgo and Huertas-Abril, 2025).

Three features distinguish this study. First, it brings together two literatures that have rarely been examined jointly, online social presence and informal second language engagement, within a single serial specification, showing how a socially populated informal environment is associated, in turn, with engagement and with self-perceived multilingual identity. Second, it treats new media literacy as a moderator and gives empirical content to its role as a boundary condition. New media literacy has been conceptualized as a set of “functional consuming, critical consuming, functional prosuming and critical prosuming” competencies (Lee et al., 2015; Luan et al., 2023); here all three associations originating in vlog learning were stronger among more digitally literate learners. Third, and methodologically distinctive, careful careless response screening was statistically consequential. When 27 complete straightliners were retained, the interactions of vlog learning with digital literacy were non significant for two of the three associations, whereas the cleaned dataset showed significant moderation throughout. In survey research on digital learning, then, data screening decisions can correspond to which boundary conditions are statistically observable.

Constraints of multilingual short video environments. The associations reported here should not be read as an unqualified endorsement of informal vlog learning. Short video platforms are not neutral learning environments: algorithmic personalization filters and prioritizes what users encounter, which may increase perceived relevance while raising concerns about autonomy, privacy, and limited information diversity (Eg et al., 2023). Platform infrastructures are also shaped by commercial, financial, and regulatory logics rather than educational purposes alone (Narayan, 2024). In multilingual short-video environments, these platform logics may also contribute to unequal visibility, as some languages, accents, creators, or multilingual repertoires may become more searchable, recommendable, or interactionally rewarded than others. In addition, algorithmic feeds are identity-relevant because users may interpret them as more or less responsive to their identities and goals (Taylor and Choi, 2022). These constraints qualify the interpretation of the findings: digital literacy matters not only as operational competence but also as a critical capacity to evaluate platform curation, content credibility, commercial persuasion, and privacy risks in multilingual digital spaces.

Theoretically, the findings give empirical content to the ecological proposition that the value of an affordance rich environment is conditional on learner agency and capability. That every association originating in vlog learning was stronger at higher levels of digital literacy is consistent with the view that, given that “affordance and agency are mutually dependent” (Jiang and Zhang, 2019; Li and Ding, 2026), an affordance rich environment is associated with different profiles of the criterion variable for differently capable learners. The study also extends the social presence and engagement traditions, which were developed largely for formal online courses (Garrison, 2007; Kreijns et al., 2022), into informal, learner driven short video settings, indicating that these constructs remain coherent and interrelated outside instructed contexts. And it locates self-perceived multilingual identity, typically studied in classrooms and through qualitative methods (Fisher et al., 2018; Forbes et al., 2021), within a quantitative model of everyday digital practice, consistent with conceptions of identity as relational and continually negotiated (Bucholtz and Hall, 2005; Darvin and Norton, 2015).

Practically, and with the associational nature of the findings kept in view, the results offer several considerations for educators and designers of informal and semi formal learning environments. Given that self-perceived multilingual identity was associated with vlog learning largely through social presence and engagement, environments that feel socially populated and that invite genuine investment may be the ones in which informal vlog consumption co occurs with identity related self-perception; features associated with a sense of co presence and immediacy (Wei et al., 2012) and with affective, cognitive, and linguistic engagement (Arndt, 2023) therefore merit attention. Given that the associations were stronger among more digitally literate learners, attention to learners’ capacity to “perceive, use, or even shape affordances” (Tong et al., 2024), namely their ability to search, evaluate, and produce multimodal content, may be a useful complement to informal exposure itself. Finally, the screening finding carries a practical lesson for researchers: routine intra individual response variability checks are advisable before modeling survey data on digital learning.

More concretely, platform features that may matter include visible and threaded comment replies, danmu or timed comments that signal co-viewing, pinned creator responses, follower or learning community spaces, curated multilingual playlists, transcript or subtitle tools, and lightweight tasks that ask learners to reuse vlog-encountered expressions. Targeted digital literacy support may also include short modules on evaluating source reliability, recognising algorithmic curation and persuasion, using subtitle, translation, and search tools effectively, and producing rather than only consuming multilingual content (Luan et al., 2023; Tong et al., 2024; Yang et al., 2022).

Several limitations qualify these conclusions. First, and most importantly, the design was cross sectional and the data self reported; the analyses therefore describe patterns of association and conditional indirect relations, and they cannot establish temporal order or directionality among vlog learning, social presence, engagement, digital literacy, and multilingual identity. The serial ordering was specified on theoretical grounds (Hayes, 2022), but reverse and reciprocal orderings are equally compatible with cross sectional data; longitudinal and experimental or quasi experimental designs are needed to examine directionality. Second, although the common method diagnostics were unremarkable, the exclusive reliance on self report leaves open the possible contribution of method variance; future work could combine self report with behavioral traces such as viewing, commenting, and production logs. Third, the sample comprised Chinese university language learners on short video platforms, so the patterns may not generalize to other ages, regions, or platforms, and cross cultural and cross platform replication would clarify the boundaries of the model. Fourth, missing values were addressed through model based imputation; although the rate was low and the statistical pattern was stable in a complete case re analysis, alternative missing data assumptions could be examined. Finally, the constructs were modeled at the composite level; future research could examine the dimension level structure (for example, co presence versus immediacy, or evaluation versus emotion) and consider which facets are most strongly associated with one another. Mixed method work would also enrich the quantitative associations reported here by illuminating how learners themselves describe the relationship between informal vlog practice and their sense of themselves as multilingual.

Although the expanded control models included socioeconomic status, dialect background, overseas experience, and academic major, intercultural contact was indexed only indirectly by overseas experience, and dialect background is at best a proxy for a multilingual family environment rather than a direct measure of it. Residual omitted-variable bias from these and other unmeasured factors therefore cannot be excluded.

Digital literacy was modelled solely as a boundary condition. Although this follows from the ecological logic that capability conditions the uptake of affordances, the construct could plausibly also be specified as an antecedent, mediator, or component of skilled informal learning; cross-sectional data cannot distinguish these specifications (Hayes, 2022; Li and Ding, 2026).

Although vlogs were treated as multilingual environments, the study did not measure the linguistic properties of the content each participant actually consumed, including which languages they viewed, the degree of multilinguality, or whether bilingual subtitles and code-switching were present. Content-analytic or log-based work characterising the material learners actually consume would be a valuable complement (Sun et al., 2026).

Relatedly, self-perceived multilingual identity was assessed as an individual difference through self-report; although the study frames identity as relational and negotiated, the measure does not capture the *in situ*, interactional positioning through which identities are co-constructed, and future work could pair such scales with discourse or ethnographic data (Bucholtz and Hall, 2005; Ou et al., 2024).

## 6 Conclusion

This study examined how short video vlog assisted informal digital language learning is associated with self-perceived multilingual identity among university learners, and whether social presence and learning engagement are modeled as serial intermediate variables and digital literacy as a moderating condition. Across 648 valid cases, vlog assisted informal learning was positively associated with self-perceived multilingual identity both directly and through an ordered social to cognitive sequence of associations running from social presence to learning engagement, and all three associations originating in vlog learning were stronger among more digitally literate learners. The measurement model was sound, the structural pattern was robust across alternative specifications, and all 12 hypotheses received statistical support. Beyond these substantive patterns, the study underscores a methodological point: removing a small number of careless responses corresponded to a different pattern of statistically detectable moderation, a reminder of the value of careful data screening. Interpreted within an ecological, affordance and agency perspective, the findings are consistent with the interpretation that an affordance rich informal environment is associated with stronger identity related self-perception to the extent that it is experienced as socially present and engaging, and to the extent that learners possess the digital literacy to act on what such environments make available. While the cross sectional design precludes claims about directionality, the results provide a theoretically grounded and quantitatively specified account of how everyday digital language practice and self-perceived multilingual identity are interrelated, and they point toward longitudinal, behavioral, and cross context research as natural next steps.

## Data Availability

The raw data supporting the conclusions of this article will be made available by the authors, without undue reservation.
