# Supplementary material for: Media consumption and self-perceived multilingual identity in short-video vlog-assisted informal digital language learning
Source: Front Psychol. 2026 Jul 15;17:1915495. doi: 10.3389/fpsyg.2026.1915495 (PMC13416255; doi:10.3389/fpsyg.2026.1915495)
Supplement: Supplementary file 1 [file Data_Sheet_1.pdf]

Supplementary Material

1 S1. Measurement instrument: structure, sources, and response formats

Five focal constructs were measured with adapted, previously validated multi-item scales, each contextualised to short-video vlog environments. All used seven-point Likert scales. The independent variable used a frequency scale (1 = never to 7 = always); social presence, learning engagement, self-perceived multilingual identity, and digital literacy used an agreement scale (1 = strongly disagree to 7 = strongly agree). Higher scores indicate higher levels of each construct.

Table S1. Construct structure, sub-dimensions, item counts, and source scales

Construct (code)	Sub-dimensions	Items	Source scale	Cronbach's α
VAILL (X)	Content consumption; interactive participation; language application	13	VAILL-Use (Sun et al., 2026)	0.958
Social presence (M1)	Co-presence; intimacy; immediacy	12	Social Presence (Wei et al., 2012; Gao et al., 2024)	0.950
Learning engagement (M2)	Affective; cognitive; linguistic	9	ISLE (Arndt, 2023)	0.933
Self-perceived multilingual identity (Y)	Evaluation; experience; emotion	15	3Es multilingual identity (Shi & Xu, 2025)	0.955
Digital literacy (W)	Access & operation; multimodal understanding; critical evaluation; participation & creation	12	English New Media Literacy (Luan et al., 2023)	0.955

Note. *The behavioural-engagement factor of the original ISLE instrument was excluded to avoid conceptual overlap with the independent variable. Three items (M1_4R, M1_8R, Y_15R) are negatively worded and were reverse-coded before scoring.*

2 S2. Item wording (bilingual)

The questionnaire was administered in Chinese; the English wording below is the parallel version used during adaptation. To avoid duplication, this section provides the full 61-item bilingual instrument rather than a shorter representative-item list. Item codes correspond to the variable names in the analytic dataset; R-suffixed items were negatively worded and reverse-coded before scoring.

S2.1. Short-video vlog-assisted informal digital language learning (X)

Source: VAILL-Use scale adapted from Sun et al. (2026). Response format: 1 = never to 7 = always.

Item	Dimension	Item wording (English)	中文条目(Chinese)
X_1	Content consumption	I watch short-video vlogs that use the target language or multiple languages.	我会观看使用目标语言或多种语言的短视频 vlog。
X_2	Content consumption	I listen to target-language or multilingual expressions in short-video vlogs.	我会收听短视频 vlog 中的目标语言或多语表达。
X_3	Content consumption	I like short-video vlogs related to language learning, cultural understanding, or multilingual life.	我会点赞与语言学习、文化理解或多语生活相关的短视频 vlog。
X_4	Content consumption	I save short-video vlogs that are useful for learning the target language or multiple languages.	我会收藏对我学习目标语言或多种语言有帮助的短视频 vlog。
X_5	Content consumption	I follow vloggers who create content in the target language or multiple languages.	我会关注使用目标语言或多种语言创作内容的 vlog 博主。
X_6	Interactive participation	After watching short-video vlogs, I interact with vloggers through comments, danmu/bullet comments, or direct messages.	看完短视频 vlog 后，我会通过评论、弹幕或私信与博主互动。
X_7	Interactive participation	After watching short-video vlogs, I interact with other viewers or learners.	看完短视频 vlog 后，我会与其他观众或学习者互动。
X_8	Interactive	I share useful target-language or multilingual short-video vlogs with friends,	我会把有价值的目标语言或多语短视频 vlog 分享给朋

Item	Dimension	Item wording (English)	中文条目(Chinese)
	participation	classmates, or online communities.	友、同学或线上社群。
X_9	Interactive participation	I participate in online discussions about vlog content, language expressions, or cultural topics.	我会参与与短视频 vlog 内容、语言表达或文化主题相关的线上讨论。
X_10	Interactive participation	I use comments, danmu/bullet comments, or hashtags to understand other learners' views on vlog content.	我会通过评论区、弹幕或话题标签了解其他学习者对 vlog 内容的看法。
X_11	Language application	I record, screenshot, or save useful vocabulary, expressions, subtitles, or sentence patterns from short-video vlogs.	我会记录、截图或保存短视频 vlog 中有用的词汇、表达、字幕或句式。
X_12	Language application	I review language expressions that I encounter in short-video vlogs.	我会复习短视频 vlog 中出现过的语言表达。
X_13	Language application	I try to use expressions learned from short-video vlogs in daily communication, writing, comments, or other learning activities.	我会在日常交流、写作、评论或其他学习活动中尝试使用短视频 vlog 中学到的表达。

S2.2. Social presence (M1)

Source: Social Presence scale adapted from Wei et al. (2012) and Gao et al. (2024). Response format: 1 = strongly disagree to 7 = strongly agree.

**Item**	**Dimension**	**Item wording (English)**	**中文条目(Chinese)**
M1_1	Co-presence	When learning languages through short-video vlogs, I feel that I am not learning alone.	通过短视频 vlog 学习语言时，我能感觉到自己并不是一个人在学习。
M1_2	Co-presence	Comments, danmu/bullet comments, likes, or shares make me feel that other learners are engaging with the same content.	评论、弹幕、点赞或转发让我感到有其他学习者和我一起关注这些内容。
M1_3	Co-presence	I can feel the real presence of vloggers or other viewers in this short-video learning space.	我能感受到博主或其他观众在这个短视频学习空间中的真实存在。
M1_4R	Co-presence	When learning languages through short-video vlogs, I feel isolated.	通过短视频 vlog 学习语言时，我觉得这个过程很孤立。
M1_5	Intimacy	I feel a sense of closeness with vloggers or other learners in short-video vlogs.	我觉得短视频 vlog 中的博主或其他学习者与我有一定的亲近感。
M1_6	Intimacy	The interactive atmosphere of short-video vlogs feels friendly and relaxed to me.	短视频 vlog 的互动氛围让我感到友好、轻松。
M1_7	Intimacy	I can sense a learning-community atmosphere in the comments, danmu/bullet comments, or related communities of short-video vlogs.	我能在短视频 vlog 的评论区、弹幕或相关社群中感受到学习共同体氛围。
M1_8R	Intimacy	Interactions in short-video vlogs make me feel distant and disconnected.	短视频 vlog 中的互动让我觉得冷漠、疏离。
M1_9	Immediacy	Comments, danmu/bullet comments, or vlogger replies make the interaction feel immediate.	评论、弹幕或博主回复让我感到互动是及时的。
M1_10	Immediacy	When I comment or ask questions under short-video vlogs, I feel that I may be noticed or responded to.	当我在短视频 vlog 下评论或提问时，我觉得自己有可能被看见或回应。
M1_11	Immediacy	In short-video vlog interactions, I feel that my views or learning needs are respected.	在短视频 vlog 的互动中，我觉得自己的观点或学习需求受到尊重。
M1_12	Immediacy	The interactive features of short-video platforms encourage me to communicate with vloggers or other learners.	短视频平台的互动功能鼓励我与博主或其他学习者交流。

S2.3. Learning engagement (M2)

Source: Informal Second Language Engagement questionnaire adapted from Arndt (2023). Response format: 1 = strongly disagree to 7 = strongly agree.

**Item**	**Dimension**	**Item wording (English)**	**中文条目(Chinese)**
M2_1	Affective engagement	I find it interesting to learn the target language or multiple languages through short-video vlogs.	我觉得通过短视频 vlog 学习目标语言或多种语言是有趣的。
M2_2	Affective engagement	Learning languages through short-video vlogs is enjoyable for me.	通过短视频 vlog 学习语言让我感到愉快。
M2_3	Affective engagement	Short-video vlogs stimulate my interest in continuing to engage with the target language or multiple languages.	短视频 vlog 激发了我继续接触目标语言或多种语言的兴趣。
M2_4	Cognitive engagement	When watching short-video vlogs, I concentrate on understanding the content.	观看短视频 vlog 时，我会集中注意力理解内容。
M2_5	Cognitive engagement	I make an effort to understand the main meanings expressed in short-video vlogs.	我会努力理解短视频 vlog 中表达的主要意思。
M2_6	Cognitive engagement	I think about how short-video vlog content relates to my language learning, cultural experiences, or identity.	我会思考短视频 vlog 内容与自己的语言学习、文化

Item	Dimension	Item wording (English)	中文条目(Chinese)
			经验或身份认同之间的关系。
M2_7	Linguistic engagement	When watching short-video vlogs, I pay special attention to vocabulary, phrases, subtitles, pronunciation, or sentence patterns.	观看短视频 vlog 时，我会特别注意其中的词汇、短语、字幕、发音或句式。
M2_8	Linguistic engagement	I compare differences among languages, accents, expressions, or cultural contexts in short-video vlogs.	我会比较短视频 vlog 中不同语言、口音、表达方式或文化语境之间的差异。
M2_9	Linguistic engagement	I try to turn expressions learned from short-video vlogs into language resources that I can use myself.	我会尝试把短视频 vlog 中学到的表达转化为自己可以使用的语言资源。

S2.4. Self-perceived multilingual identity (Y)

Source: 3Es multilingual identity scale adapted from Shi and Xu (2025). Response format: 1 = strongly disagree to 7 = strongly agree.

Item	Dimension	Item wording (English)	中文条目(Chinese)
Y_1	Evaluation	I regard being able to understand or use multiple languages as part of my identity.	我把能够理解或使用多种语言看作自我身份的一部分。
Y_2	Evaluation	I can imagine myself becoming a person who communicates in multiple languages in the future.	我能想象自己未来成为一个能够使用多种语言进行交流的人。
Y_3	Evaluation	For me, developing multilingual abilities is important for personal growth, learning, or future career development.	对我来说，发展多语能力对个人成长、学习或未来职业很重要。
Y_4	Evaluation	Learning multiple languages helps me view issues from different perspectives.	学习多种语言有助于我从不同角度看待问题。
Y_5	Evaluation	Learning a new language helps me better understand the languages I already know.	学习一种新语言能帮助我更好地理解自己已经掌握的语言。
Y_6	Evaluation	Engaging with multiple languages helps me understand other cultures and better understand my own culture.	接触多种语言有助于我理解不同文化，也更好地理解自己的文化。
Y_7	Experience	I actively look for opportunities to use the languages I am learning or already know, either online or offline.	我会主动寻找机会在线上或线下使用自己正在学习或已经掌握的语言。
Y_8	Experience	Short-video vlogs expose me to different language communities, cultural groups, or transnational lifestyles.	短视频 vlog 让我接触到不同语言群体、文化群体或跨国生活方式。
Y_9	Experience	In daily communication or digital platform interactions, I sometimes naturally mix or switch between different languages.	在日常交流或数字平台互动中，我有时会自然地混合或切换不同语言。
Y_10	Experience	Vloggers, friends, classmates, or online communities encourage me to continue learning and using multiple languages.	博主、朋友、同学或线上社群会鼓励我继续学习和使用多种语言。
Y_11	Experience	I feel connected to certain multilingual learning communities, intercultural communities, or digital language communities.	我觉得自己与某些多语学习社群、跨文化社群或数字语言社群有联系。
Y_12	Emotion	Learning or using multiple languages makes me feel happy.	学习或使用多种语言让我感到愉快。
Y_13	Emotion	I feel proud when I can understand or use multiple languages.	当我能理解或使用多种语言时，我会感到自豪。
Y_14	Emotion	I believe that I can continue developing my multilingual abilities.	我相信自己能够持续发展多语能力。
Y_15R	Emotion	Learning or using multiple languages often makes me feel anxious, frustrated, or excluded.	学习或使用多种语言常常让我感到焦虑、挫败或排斥。

S2.5. Digital literacy (W)

Source: English New Media Literacy scale adapted from Luan et al. (2023). Response format: 1 = strongly disagree to 7 = strongly agree.

Item	Dimension	Item wording (English)	中文条目(Chinese)
W_1	Digital access and operation	I can effectively search for short-video vlogs that suit my language-learning goals.	我能有效搜索到适合自己语言学习目标的短视频 vlog。
W_2	Digital access and operation	I can skillfully use subtitles, search, saving, playback speed, translation, hashtags, or recommendation features on short-video platforms to support language learning.	我能熟练使用短视频平台的字幕、搜索、收藏、倍速、翻译、话题标签或推荐功能来支持语言学习。
W_3	Digital access and operation	I know how to protect my privacy and account security on short-video platforms.	我知道如何在短视频平台上保护自己的隐私和账号安全。
W_4	Multimodal understanding	I can understand how text, speech, subtitles, images, emojis, and background music jointly convey meaning in short-video vlogs.	我能理解短视频 vlog 中文字、语音、字幕、图像、表情和背景音乐共同传递的意义。
W_5	Multimodal understanding	I can distinguish facts, opinions, personal experiences, and commercial promotion in short-video vlogs.	我能区分短视频 vlog 中的事实、观点、个人经验和商业推广。
W_6	Multimodal understanding	I compare information from short-video vlogs with other sources to judge its accuracy.	我会把短视频 vlog 中的信息与其他来源进行比

Item	Dimension	Item wording (English)	中文条目(Chinese)
			较，以判断其准确性。
W_7	Critical evaluation	I can judge whether language expressions, cultural explanations, or learning advice in short-video vlogs are reliable.	我能判断短视频 vlog 中的语言表达、文化解释或学习建议是否可靠。
W_8	Critical evaluation	I can identify possible stereotypes, biases, exaggerations, or algorithmic persuasion in short-video vlogs.	我能识别短视频 vlog 中可能存在的刻板印象、偏见、夸大表达或算法诱导。
W_9	Critical evaluation	I can judge whether a short-video vlog is truly suitable for my language-learning goals.	我能判断某个短视频 vlog 是否真正适合我的语言学习目标。
W_10	Participation and creation	I can express my views responsibly in comments, danmu/bullet comments, or shares.	我能在评论、弹幕或分享中负责任地表达自己的观点。
W_11	Participation and creation	I can use short-video vlogs to create learning notes, subtitle summaries, expression lists, or my own language-learning content.	我能利用短视频 vlog 制作学习笔记、字幕整理、表达清单或自己的语言学习内容。
W_12	Participation and creation	I can adjust my digital language-learning practices based on comments, feedback, or platform data.	我能根据评论、反馈或平台数据调整自己的数字语言学习方式。

Note. The bilingual item wording is shown in administration order. This full table replaces the earlier representative-item list to avoid duplication across the supplement.

3 S3. Item-level descriptive and psychometric statistics

For each item the table reports the mean (M), standard deviation (SD), skewness, excess kurtosis, the pre-imputation missing rate among the 648 valid cases, the corrected item–total (item–rest) correlation within its construct, and the standardized loading (λ) from a single-factor model of that construct. All items were approximately normally distributed (|skewness| ≤ 0.3, |kurtosis| ≤ 0.72, within the |skewness| < 2 and |kurtosis| < 7 guidelines for maximum-likelihood estimation); all corrected item–total correlations exceeded .30 (range 0.72–0.80); all standardized loadings exceeded .50 (range 0.74–0.82); and no item exceeded 2.62% missing. These results support item retention and convergent validity.

Table S2. Item-level statistics (N = 648)

Item	Sub-dimension	M	SD	Skew	Kurt	Miss %	Item–rest r	λ
X_1	Content	4.07	1.28	0.02	-0.57	1.39	0.77	0.78
X_2	Content	3.60	1.30	0.19	-0.53	1.23	0.78	0.80
X_3	Content	4.41	1.27	-0.08	-0.58	1.23	0.79	0.81
X_4	Content	4.19	1.29	-0.10	-0.59	2.01	0.79	0.80
X_5	Content	3.62	1.26	0.07	-0.50	1.70	0.78	0.80
X_6	Interactive	4.69	1.20	-0.22	-0.41	1.08	0.77	0.79
X_7	Interactive	4.65	1.34	-0.30	-0.51	0.15	0.78	0.81
X_8	Interactive	3.98	1.17	0.08	-0.36	1.70	0.78	0.80
X_9	Interactive	3.67	1.27	0.13	-0.36	1.23	0.78	0.80
X_10	Interactive	3.66	1.34	0.19	-0.52	1.39	0.80	0.82
X_11	Lang. app.	4.24	1.26	-0.18	-0.55	1.54	0.77	0.79
X_12	Lang. app.	3.73	1.22	0.09	-0.32	0.31	0.76	0.78
X_13	Lang. app.	4.32	1.33	-0.10	-0.55	1.23	0.78	0.79
M1_1	Co-presence	3.94	1.23	0.02	-0.41	2.16	0.74	0.76
M1_2	Co-presence	3.99	1.18	-0.02	-0.28	2.01	0.73	0.75
M1_3	Co-presence	3.66	1.28	0.21	-0.33	1.85	0.78	0.80

Item	Sub-dimension	M	SD	Skew	Kurt	Miss %	Item–rest r	λ
M1_4R	Co-presence	4.26	1.27	-0.11	-0.41	0.93	0.76	0.78
M1_5	Intimacy	4.09	1.23	0.00	-0.32	2.31	0.78	0.80
M1_6	Intimacy	4.20	1.21	-0.07	-0.37	1.23	0.77	0.79
M1_7	Intimacy	3.73	1.18	0.03	-0.21	1.85	0.77	0.80
M1_8R	Intimacy	3.45	1.17	0.23	-0.27	1.23	0.73	0.75
M1_9	Immediacy	4.53	1.23	-0.10	-0.42	2.01	0.75	0.78
M1_10	Immediacy	4.59	1.27	-0.12	-0.53	2.31	0.77	0.80
M1_11	Immediacy	3.91	1.30	0.07	-0.50	1.70	0.76	0.79
M1_12	Immediacy	3.43	1.28	0.28	-0.48	1.85	0.78	0.80
M2_1	Affective	4.12	1.33	0.06	-0.65	2.01	0.77	0.80
M2_2	Affective	3.77	1.26	0.07	-0.48	1.54	0.74	0.78
M2_3	Affective	4.41	1.21	-0.15	-0.54	1.39	0.77	0.80
M2_4	Cognitive	4.38	1.27	-0.11	-0.58	1.54	0.75	0.77
M2_5	Cognitive	4.05	1.30	0.03	-0.72	0.62	0.76	0.79
M2_6	Cognitive	4.02	1.26	-0.03	-0.56	1.54	0.74	0.77
M2_7	Linguistic	3.50	1.17	0.14	-0.53	2.01	0.73	0.76
M2_8	Linguistic	4.00	1.22	0.04	-0.41	1.54	0.73	0.76
M2_9	Linguistic	3.69	1.25	0.15	-0.50	1.39	0.76	0.79
Y_1	Evaluation	4.10	1.18	0.01	-0.37	1.08	0.76	0.78
Y_2	Evaluation	4.08	1.17	-0.08	-0.35	1.39	0.74	0.76
Y_3	Evaluation	4.56	1.23	-0.13	-0.61	1.85	0.75	0.77
Y_4	Evaluation	4.34	1.18	-0.10	-0.34	1.70	0.72	0.74
Y_5	Evaluation	4.51	1.27	-0.21	-0.45	0.77	0.76	0.78
Y_6	Evaluation	4.18	1.16	0.08	-0.50	1.54	0.75	0.77
Y_7	Experience	4.43	1.14	-0.16	-0.28	1.08	0.73	0.76
Y_8	Experience	4.72	1.12	-0.24	-0.34	2.01	0.74	0.76
Y_9	Experience	4.00	1.23	0.03	-0.32	1.85	0.77	0.79
Y_10	Experience	4.46	1.18	-0.08	-0.48	1.70	0.77	0.79
Y_11	Experience	3.51	1.23	0.16	-0.40	1.70	0.76	0.78
Y_12	Emotion	4.43	1.25	0.05	-0.56	2.01	0.75	0.76
Y_13	Emotion	4.39	1.20	0.06	-0.42	1.39	0.75	0.76
Y_14	Emotion	4.20	1.22	0.08	-0.47	2.62	0.72	0.74
Y_15R	Emotion	4.71	1.15	-0.11	-0.32	0.93	0.74	0.76
W_1	Access/op.	3.86	1.38	0.09	-0.52	1.39	0.78	0.80
W_2	Access/op.	3.83	1.26	0.11	-0.55	1.39	0.79	0.81
W_3	Access/op.	4.71	1.27	-0.24	-0.52	1.39	0.76	0.78

Item	Sub-dimension	M	SD	Skew	Kurt	Miss %	Item–rest r	λ
W_4	Multimodal	4.10	1.26	-0.07	-0.55	0.93	0.78	0.80
W_5	Multimodal	3.96	1.28	0.01	-0.61	1.23	0.78	0.80
W_6	Multimodal	4.37	1.32	-0.13	-0.51	1.70	0.78	0.80
W_7	Critical eval.	4.61	1.26	-0.18	-0.42	1.08	0.79	0.81
W_8	Critical eval.	3.50	1.26	0.20	-0.54	1.08	0.77	0.79
W_9	Critical eval.	4.73	1.32	-0.20	-0.65	1.85	0.79	0.81
W_10	Participation	4.29	1.32	-0.16	-0.37	1.39	0.76	0.78
W_11	Participation	3.41	1.28	0.23	-0.45	1.39	0.77	0.79
W_12	Participation	4.13	1.27	-0.01	-0.49	1.39	0.80	0.82

Note. *Skew = skewness; Kurt = excess kurtosis; Miss % = pre-imputation missing rate among the 648 valid cases; Item–rest r = corrected item–total correlation within the construct; λ = standardized loading from a single-factor model of the construct. R-suffixed items were reverse-coded before analysis. Shaded rows mark the first item of each construct.*

4 S4. Control and background variables

Three variables were entered as statistical controls in the main analyses: self-rated target-language proficiency, number of foreign languages learned, and daily short-video platform-use intensity. Four further background variables (socioeconomic status, overseas experience, dialect background, and academic major) were collected and used in the expanded-controls sensitivity model (Section S13). Full demographic characteristics are reported in Table 1 of the main text.

Table S3. Control and background variables (N = 648)

Variable (role)	Coding	Descriptive statistics
Self-rated target-language proficiency (control)	1 = very low … 7 = very high	M = 3.50, SD = 1.28, range 1–7
Number of foreign languages learned (control)	count (excl. mother tongue/dialects)	M = 1.48, SD = 0.69, range 1–4
Daily short-video platform use (control)	1 = <30 min … 5 = >3 h	M = 3.04, SD = 1.02, range 1–5
Socioeconomic status (expanded model)	ordinal 1–5	M = 2.99, SD = 0.90, range 1–5
Overseas experience (expanded model)	0 = none, 1 / 2 = increasing	0: 485 (74.8%); 1: 125 (19.3%); 2: 38 (5.9%)
Dialect background (expanded model)	0 / 1	0: 201 (31%); 1: 447 (69%)
Academic major (expanded model)	1–6 (see Table 1)	1: 182 (28.1%); 2: 136 (21%); 3: 139 (21.5%); 4: 97 (15%); 5: 63 (9.7%); 6: 31 (4.8%)
Age (years)	continuous	M = 20.63, SD = 1.46, range 18–25

Note. *The first three rows are the statistical controls used in all models; the remaining rows are background variables used only in the expanded-controls sensitivity analysis (Section S13). Category labels for academic major follow Table 1.*

5 S5. Careless-response screening

The raw dataset contained 675 responses to 61 focal items. Careless or insufficient-effort responding was screened using an intra-individual response-variability (longstring) criterion: for each respondent the standard deviation across all 61 items was computed, and a value of zero indicates complete straightlining (an identical option for every item). 27 respondents (4.0%) met this criterion, each having entered the same response across all 61 items (within-respondent SD = 0, maximum longstring = 61). Because such records carry no usable covariance information and inflate internal consistency and bivariate associations, all 27 were removed, yielding the valid analytic sample of N = 648. As reported in the main text, retaining these records masked two of the three moderation effects, so their removal was consequential rather than cosmetic.

6 S6. Missing data and tests of missingness mechanism

Among the 648 valid cases, item-level missingness was low: 590 missing values across 39,528 item responses (648 cases × 61 items), or 1.49%, with no item exceeding 2.62% missing (per-item rates appear in Table S2). Because missing values were scattered, 390 of the 648 cases (60.2%) had at least one missing item, so listwise deletion would have discarded the majority of the sample.

Two diagnostics were consistent with data missing completely at random (MCAR). Little's MCAR test was non-significant, $\chi^2(12{,}221) = 12{,}278.38$, $p = 0.355$. A logistic regression predicting whether a case had any missing value from observed background and study variables was also non-significant (likelihood-ratio $\chi^2(22) = 27.55$, $p = 0.19$), indicating no detectable dependence of missingness on observed covariates. Missing values were therefore imputed at the item level using an expectation-maximization-based iterative (regression) imputer, with imputed values constrained to the 1–7 response range; under MCAR (and more generally MAR) this estimator yields consistent estimates, and the substantive findings were unchanged in a complete-case re-analysis of the 258 fully observed cases (Section S13).

Note. *Denominator uses the valid analytic sample (648 × 61 = 39,528). Little's test was evaluated across 208 distinct missingness patterns.*

7 S7. Parcelling: rationale and construction

Because the five constructs are measured by 61 items, a fully item-level structural model would estimate many parameters relative to the sample. To obtain a stable, well-conditioned measurement model while preserving the theorised multidimensional structure, the confirmatory factor analysis used the 16 first-order sub-dimensions as domain-representative parcels. Parcels were constructed transparently and theoretically rather than to maximise fit: each parcel is the unweighted mean of the items in one a priori sub-dimension. The item-level model (Section S8) corroborates the parcel solution.

Table S4. Composition of the 16 dimension-level parcels

Construct	Sub-dimension (parcel)	Items	k
VAILL	Content consumption	X_1, X_2, X_3, X_4, X_5	5
VAILL	Interactive participation	X_6, X_7, X_8, X_9, X_10	5
VAILL	Language application	X_11, X_12, X_13	3
Social presence	Co-presence	M1_1, M1_2, M1_3, M1_4R	4
Social presence	Intimacy	M1_5, M1_6, M1_7, M1_8R	4
Social presence	Immediacy	M1_9, M1_10, M1_11, M1_12	4
Learning engagement	Affective	M2_1, M2_2, M2_3	3
Learning engagement	Cognitive	M2_4, M2_5, M2_6	3
Learning engagement	Linguistic	M2_7, M2_8, M2_9	3
Self-perceived multilingual identity	Evaluation	Y_1, Y_2, Y_3, Y_4, Y_5, Y_6	6
Self-perceived multilingual identity	Experience	Y_7, Y_8, Y_9, Y_10, Y_11	5
Self-perceived multilingual identity	Emotion	Y_12, Y_13, Y_14, Y_15R	4
Digital literacy	Access & operation	W_1, W_2, W_3	3
Digital literacy	Multimodal understanding	W_4, W_5, W_6	3
Digital literacy	Critical evaluation	W_7, W_8, W_9	3
Digital literacy	Participation & creation	W_10, W_11, W_12	3

Note. *k = number of items averaged into the parcel. The 13, 12, 9, 15, and 12 items of the five constructs reduce to 3, 3, 3, 3, and 4 parcels respectively (16 in total), each loading on its intended factor.*

8 S8. Item-level confirmatory factor analysis

To complement the parcel-level measurement model, a correlated five-factor CFA was estimated at the item level, with all 61 items loading on their intended construct (semopy; N = 648). Fit was acceptable, $\chi^2(1,759) = 4,154.32$, $\chi^2/df = 2.362$, CFI = 0.925, TLI = 0.922, RMSEA = 0.046, SRMR = 0.033, and all standardized loadings were substantial and significant (range 0.74–0.82). As expected, incremental fit indices are more demanding at the item level than at the parcel level but remain within or close to conventional thresholds, indicating that the five-factor structure holds at the item level and that the strong parcel-level fit is not an artefact of parcelling.

Table S5. Item-level CFA fit, by construct loading range

Construct	Std. loading range
VAILL (X)	0.78–0.82
Social presence (M1)	0.75–0.80
Learning engagement (M2)	0.76–0.80
Self-perceived multilingual identity (Y)	0.74–0.79
Digital literacy (W)	0.78–0.81

Table S6. Standardized item loadings from the 61-indicator five-factor model

Item	Construct	Sub-dimension	λ (5-factor)
X_1	VAILL	Content	0.784
X_2	VAILL	Content	0.797
X_3	VAILL	Content	0.805
X_4	VAILL	Content	0.802
X_5	VAILL	Content	0.797
X_6	VAILL	Interactive	0.790
X_7	VAILL	Interactive	0.806
X_8	VAILL	Interactive	0.804
X_9	VAILL	Interactive	0.804
X_10	VAILL	Interactive	0.819
X_11	VAILL	Lang. app.	0.791
X_12	VAILL	Lang. app.	0.783
X_13	VAILL	Lang. app.	0.796
M1_1	Social presence	Co-presence	0.762
M1_2	Social presence	Co-presence	0.750
M1_3	Social presence	Co-presence	0.804
M1_4R	Social presence	Co-presence	0.784
M1_5	Social presence	Intimacy	0.798
M1_6	Social presence	Intimacy	0.789
M1_7	Social presence	Intimacy	0.796
M1_8R	Social presence	Intimacy	0.752

Item	Construct	Sub-dimension	λ (5-factor)
M1_9	Social presence	Immediacy	0.777
M1_10	Social presence	Immediacy	0.793
M1_11	Social presence	Immediacy	0.786
M1_12	Social presence	Immediacy	0.796
M2_1	Learning engagement	Affective	0.801
M2_2	Learning engagement	Affective	0.782
M2_3	Learning engagement	Affective	0.801
M2_4	Learning engagement	Cognitive	0.774
M2_5	Learning engagement	Cognitive	0.790
M2_6	Learning engagement	Cognitive	0.765
M2_7	Learning engagement	Linguistic	0.762
M2_8	Learning engagement	Linguistic	0.761
M2_9	Learning engagement	Linguistic	0.788
Y_1	Self-perceived multilingual identity	Evaluation	0.777
Y_2	Self-perceived multilingual identity	Evaluation	0.756
Y_3	Self-perceived multilingual identity	Evaluation	0.763
Y_4	Self-perceived multilingual identity	Evaluation	0.744
Y_5	Self-perceived multilingual identity	Evaluation	0.780
Y_6	Self-perceived multilingual identity	Evaluation	0.770
Y_7	Self-perceived multilingual identity	Experience	0.756
Y_8	Self-perceived multilingual identity	Experience	0.755
Y_9	Self-perceived multilingual identity	Experience	0.788
Y_10	Self-perceived multilingual identity	Experience	0.789
Y_11	Self-perceived multilingual identity	Experience	0.784
Y_12	Self-perceived multilingual identity	Emotion	0.763
Y_13	Self-perceived multilingual identity	Emotion	0.766
Y_14	Self-perceived multilingual identity	Emotion	0.742
Y_15R	Self-perceived multilingual identity	Emotion	0.762
W_1	Digital literacy	Access/op.	0.800
W_2	Digital literacy	Access/op.	0.807
W_3	Digital literacy	Access/op.	0.784
W_4	Digital literacy	Multimodal	0.801
W_5	Digital literacy	Multimodal	0.804
W_6	Digital literacy	Multimodal	0.802
W_7	Digital literacy	Critical eval.	0.814
W_8	Digital literacy	Critical eval.	0.790

Item	Construct	Sub-dimension	λ (5-factor)
W_9	Digital literacy	Critical eval.	0.808
W_10	Digital literacy	Participation	0.779
W_11	Digital literacy	Participation	0.793
W_12	Digital literacy	Participation	0.814

Note. *All loadings $p < .001$. Shaded rows mark the first item of each construct.*

9 S9. Reliability and convergent validity

All scales demonstrated excellent internal consistency and convergent validity. Cronbach's α ranged from 0.933 to 0.958, McDonald's ω from 0.944 to 0.963, and composite reliability (CR) from 0.944 to 0.963; average variance extracted (AVE) ranged from 0.615 to 0.670, exceeding the .50 threshold for every construct.

Table S7. Reliability and convergent validity

Construct	Items	α	ω	CR	AVE
VAILL (X)	13	0.958	0.963	0.963	0.665
Social presence (M1)	12	0.950	0.956	0.956	0.644
Learning engagement (M2)	9	0.933	0.944	0.944	0.653
Self-perceived multilingual identity (Y)	15	0.955	0.960	0.960	0.615
Digital literacy (W)	12	0.955	0.961	0.961	0.670

Note. *α = Cronbach's alpha; ω = McDonald's omega; CR = composite reliability; AVE = average variance extracted.*

10 S10. Discriminant validity

Discriminant validity was supported by the Fornell–Larcker criterion (the square root of AVE exceeded each construct's correlations with all others; see main-text Table 2) and by the heterotrait–monotrait ratios below, all of which fell well under the conservative .85 threshold (maximum = .712).

Table S8. Heterotrait–monotrait ratios (HTMT)

	X	M1	M2	Y	W
X	—				
M1	0.544	—			
M2	0.584	0.665	—		
Y	0.588	0.696	0.712	—	
W	0.260	0.541	0.499	0.517	—

To test the two most closely related boundaries directly, nested confirmatory models were compared with chi-square difference tests.

Table S9. Nested-model discriminant tests

Test	Constrained model	Freed model	$\Delta\chi^2$ (df)	p
Social presence vs learning engagement (parcels)	1 factor: CFI 0.932	5 factors: CFI 0.998	567.2 (4)	< .001
Language application vs linguistic engagement (items)	1 factor: CFI 0.694	2 factors: CFI 1.000	650.8 (1)	< .001

Note. *In the item-level test the two factors were correlated but distinct (r = 0.52). Both comparisons favour the distinct-factor specification.*

11 S11. Common method variance

Three diagnostics were examined, while recognising that none can establish the absence of common method variance. Harman's single-factor test showed that the first unrotated factor accounted for 40.80% of the variance (below 50%). Full-collinearity variance inflation factors (VIFs) were all below the 3.3 criterion (Table S10). A confirmatory single-factor model, forcing all 16 parcels onto one common factor, fit the data poorly (CFI = 0.626, TLI = 0.568, RMSEA = 0.217), far worse than the five-factor model (CFI = 0.998), indicating that a single common factor cannot reproduce the observed structure. Together these diagnostics suggest that common method variance is unlikely to account for the findings, but they do not rule it out; because all constructs were self-reported at one time point, some method influence remains possible.

Table S10. Full-collinearity VIFs

Construct	VIF
VAILL	1.67
Social presence	2.19
Learning engagement	2.21
Self-perceived multilingual identity	2.42
Digital literacy	1.50

Note. *VIFs were obtained by regressing each composite on the remaining four; all values fall below the 3.3 threshold (Kock, 2015).*

12 S12. Moderation: effect sizes and conditional indirect statistical associations

Because statistical significance is readily attained at this sample size, effect sizes are reported for each interaction. The increment to explained variance from the X × W term was small for every criterion variable, but the simple slopes show a visible gradient, steepest on the path to self-perceived multilingual identity (SD of digital literacy = 1.06).

Table S11. Interaction effect sizes and simple slopes

Criterion variable	ΔR^2	f^2	Slope at −1 SD W	Slope at +1 SD W	Ratio
Social presence (M1)	0.005	0.009	0.32	0.46	1.4×
Learning engagement (M2)	0.007	0.014	0.24	0.40	1.7×
Self-perceived multilingual identity (Y)	0.008	0.020	0.11	0.27	2.5×

Note. *$f^2 = \Delta R^2 / (1 - R^2\_full)$; values of .009–.020 are small by conventional benchmarks. Slopes are unstandardized.*

Table S12. Conditional indirect statistical associations and indices of moderated mediation (main model)

Indirect association	Low W	Mean W	High W	Index (95% CI)
X → M1 → Y	0.084 [.054, .120]	0.102 [.070, .137]	0.120 [.083, .163]	[.003, .033]
X → M2 → Y	0.063 [.037, .093]	0.084 [.059, .113]	0.106 [.074, .142]	[.007, .035]
X → M1 → M2 → Y	0.030 [.018, .045]	0.036 [.024, .051]	0.043 [.028, .061]	[.001, .012]
Total indirect	0.177 [.131, .228]	0.223 [.181, .266]	0.269 [.219, .321]	[.020, .069]

Note. *Unstandardized conditional indirect statistical associations with 95% bootstrap CIs; low/high W = ±1 SD. A CI for the index excluding zero indicates significant moderated mediation. Reproduced from main-text Table 8.*

13 S13. Robustness and sensitivity analyses

The substantive pattern was stable across control-variable sets, estimators, a complete-case re-analysis, and subgroups by language repertoire. The background-expanded model adds socioeconomic status, dialect background, overseas experience, and academic major to the three main controls; it was added in revision to address potential omitted-variable bias and leaves the conclusions unchanged.

Table S13. Direct, indirect, serial effects and X × W interactions across specifications

Specification	Direct c′	Total indirect	Serial indirect	X × W (M1 / M2 / Y)
No controls	0.171	0.337	0.077	.019 / .003 / < .001
Main controls	0.177	0.340	0.076	.015 / .003 / < .001
Expanded demographic controls	0.175	0.340	0.074	.016 / .004 / < .001
Background-expanded (SES, dialect, overseas, major) — added	0.169	0.347	0.076	.012 / .004 / < .001
SEM (ML estimator)	0.177	—	—	all paths p < .001
Complete-case (listwise, n = 258)	0.221	0.307	0.111	pattern retained
One language (n = 407)	0.183	0.338	0.077	pattern retained
Two+ languages (n = 241)	0.169	0.342	0.072	pattern retained

Note. *Estimates are unstandardized. The shaded "Background-expanded" row was computed for the revision (direct = 0.169, total indirect = 0.347, serial = 0.076; X × W p = 0.012 / 0.004 / < .001); all other rows are from the main-text analysis. Intercultural contact was indexed only indirectly (overseas experience) and dialect background is a proxy for a multilingual home environment, so residual omitted-variable bias cannot be excluded.*

14 S14. Reverse-order sensitivity analyses

Because the design is cross-sectional, two reverse-order analyses assessed whether alternative orderings are also supported by the data, using the same estimator, controls, mean-centring, and bootstrap as the main analysis. They gauge the plausibility of alternative orderings only and are not interpreted as evidence of causal direction.

Table S14. Reverse-order model summary

Model	Predictor	Mediators / criterion variable	Purpose
Main	VAILL	Social presence → engagement → self-perceived multilingual identity	Theoretically specified model
R1	Self-perceived multilingual identity	VAILL, social presence, engagement (separate criterion variable)	Reviewer's alternative explanation
R2	Self-perceived multilingual identity	Social presence → engagement → VAILL	Reversed conditional process model

Table S15. Model R1 — identity as antecedent (simple regressions; + W + controls)

Association	b	SE	p	β	Forward β
Identity → VAILL	0.600	0.041	< .001	0.54	0.47
Identity → social presence	0.563	0.035	< .001	0.53	0.41
Identity → engagement	0.619	0.036	< .001	0.57	0.47

Note. *β = standardized coefficient. Reverse associations are at least as strong as the forward associations, confirming that a reversed ordering is statistically plausible.*

Table S16. Model R2 — reversed conditional process model (regression estimates)

Criterion variable	Path	b	SE	t	p
Social presence (M1)	Identity → M1	0.558	0.035	15.84	< .001
	W → M1	0.242	0.031	7.88	< .001
	Identity × W → M1	0.040	0.026	1.55	0.123
Engagement (M2)	Identity → M2	0.457	0.041	11.09	< .001
	M1 → M2	0.284	0.039	7.25	< .001
	W → M2	0.114	0.032	3.58	< .001
	Identity × W → M2	0.019	0.026	0.74	0.458
VAILL (X)	Identity → X	0.309	0.049	6.29	< .001
	M1 → X	0.193	0.044	4.35	< .001
	M2 → X	0.272	0.043	6.31	< .001
	W → X	-0.097	0.035	-2.75	0.006
	Identity × W → X	0.115	0.028	4.05	< .001

Note. *R^2 = 0.494 (M1), 0.521 (M2), 0.457 (X). Indirect statistical associations (mean W; 5,000-bootstrap CIs): via social presence 0.108 [0.058, 0.158]; via engagement 0.124 [0.084, 0.167]; serial 0.043 [0.027, 0.062]; total indirect 0.275 [0.212, 0.340]; direct 0.309 [0.215, 0.406]. The direct and serial indirect associations remain significant under reversal; however, the indices of moderated mediation all include zero, so the moderated-mediation structure is significant only in the hypothesized direction. Because the simple and serial associations are essentially symmetric across direction, the data cannot adjudicate temporal priority.*

15 S15. Computational reproducibility

All analyses were conducted in Python. Path models were estimated by ordinary least squares (statsmodels); confirmatory factor analyses were estimated with semopy; figures were produced with matplotlib. Predictors and the moderator were mean-centred before forming product terms, and all indirect and conditional indirect statistical associations used percentile bootstrap confidence intervals based on 5,000 resamples with a fixed random seed, so the reported values are reproducible. The serial mediation model is structurally equivalent to Hayes's Model 6 and the full conditional process model to Model 85; the PROCESS macro itself was not used.

The analysis script and a machine-readable results file accompany this submission. Data are available as described in the Data Availability Statement of the main text.
